# Sp1 mechanotransduction regulates breast cancer cell invasion in engineered viscoelastic extracellular matrices

**DOI:** 10.1016/j.biomaterials.2025.123755

**Published:** 2025-10-03

**Authors:** Abhishek Sharma, Rowan F. Steger, Jen M. Li, Samuel Y. Fong, Neha Saxena, Jane A. Baude, Kellie A. Heom, Siddharth S. Dey, Ryan S. Stowers

**Affiliations:** a Department of Mechanical Engineering, University of California, Santa Barbara, Santa Barbara, CA, USA; b Department of Chemical Engineering, University of California, Santa Barbara, Santa Barbara, CA, USA; c Department of Molecular, Cellular, and Developmental Biology, University of California, Santa Barbara, Santa Barbara, CA, USA; d Department of Bioengineering, University of California, Santa Barbara, Santa Barbara, CA, USA

## Abstract

Breast cancer progression involves extensive remodeling of the extracellular matrix (ECM), including increased stiffness, altered viscoelasticity (stress relaxation), and elevated collagen levels. While in vitro experiments have revealed a role for each of these factors in individually promoting malignant behavior, their combined effects remain unclear. Here, we engineered alginate-collagen hydrogels with independently tunable stiffness, stress relaxation, and collagen density to dissect how the complex ECM environment regulates cancer cell phenotype. We show that high stiffness, fast stress relaxation, and high collagen density led to changes in cell morphology, marked by decreased roundness, and promoted spheroid invasion in both breast cancer and non-transformed mammary epithelial cells. Single cell migration speed and displacement were greatest in matrices of high stiffness, low collagen density, and slow stress relaxation. RNA-seq and Cleavage Under Targets and Tagmentation (CUT&Tag)-seq revealed that high stiffness and fast stress relaxing groups were enriched for Sp1 target gene expression as well as increased Sp1 binding at genomic loci. Notably, analysis of publicly available claudin-low breast cancer data showed that high expression of the Sp1-regulated genes in fast stress relaxing groups was correlated with significantly reduced patient survival. Mechanistically, we found that phosphorylated Sp1 (T453) exhibited increased nuclear localization in matrices with high stiffness and fast stress relaxation. Furthermore, Sp1 phosphorylation was regulated by PI3K and ERK1/2 activity, as well as actomyosin contractility. Our tunable hydrogel platform reveals that multiple tumor-mimicking cues within complex viscoelastic microenvironments reinforce malignant traits, with Sp1 acting as a mechanoresponsive transcription factor that transduces these signals.

## 1. Introduction

**Fig. 1.**
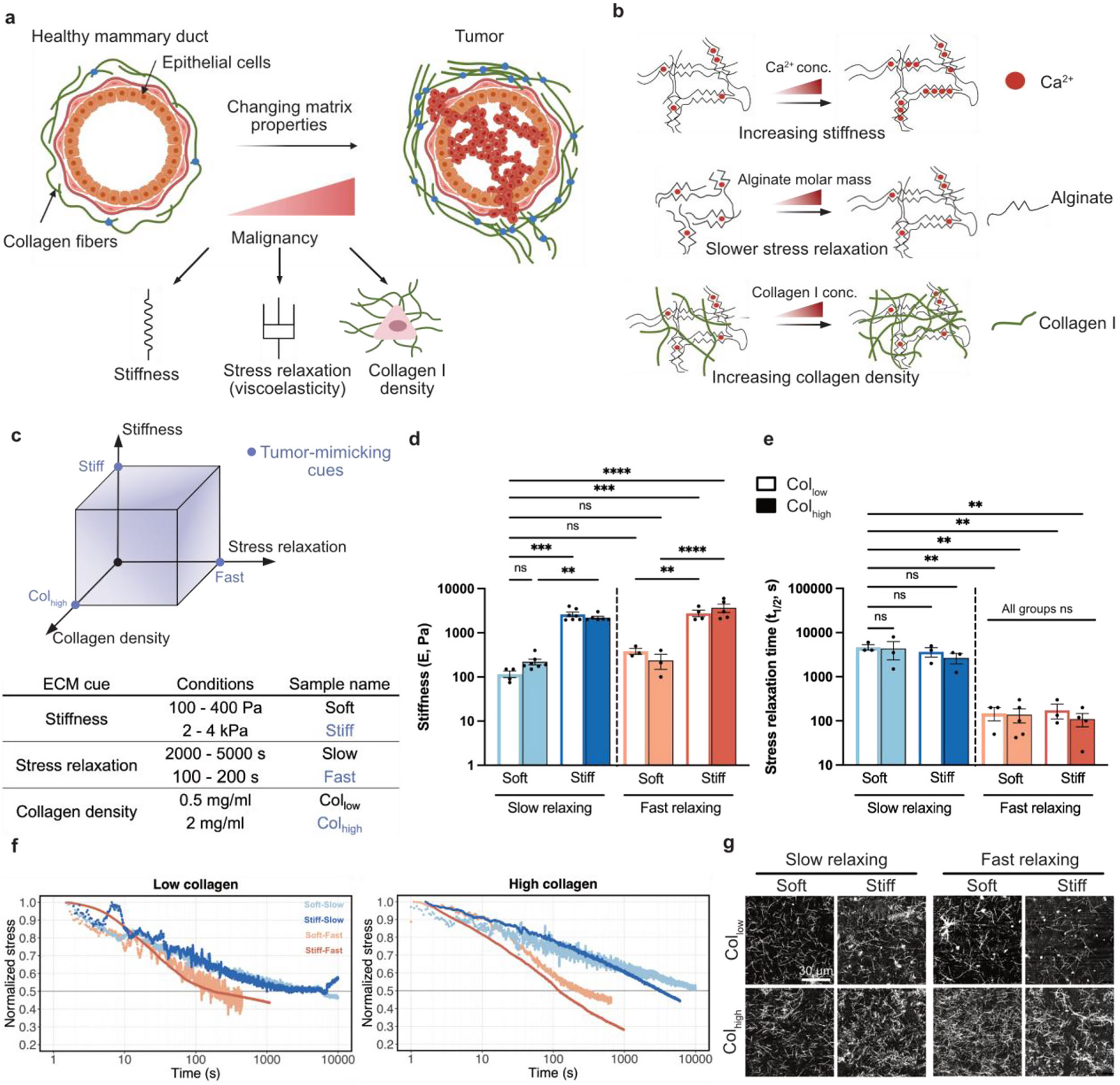
Independent tuning of stiffness, stress relaxation, and collagen fiber density with alginate-collagen I matrices. **a,** Breast cancer progression is accompanied by changes in ECM properties such as elevated stiffness, altered stress relaxation, and increased production of collagen I. **b,** Alginate-collagen matrices allow for independent tuning of stiffness and stress relaxation by varying the calcium ion concentration and the molar mass of the alginate chains respectively. Further, varying amounts of collagen can be incorporated as interpenetrating networks in these matrices. **c,** We developed 8 matrix conditions as combinations of soft or stiff, slow relaxing or fast relaxing, and low or high collagen density. The table describes the nomenclature used throughout the study. **d,** Stiffness measurement of soft and stiff alginate-collagen matrices. **e,** Measurement of the time taken to relax stress to half of the maximum value under constant 10 % strain. For both **d, e,** n ≥ 3; mean ± s. e.m.; ANOVA followed by followed by post-hoc Sidak’s multiple comparison test was performed. **f,** Stress relaxation curves for representative alginate-collagen matrices with low and high collagen density. **g,** Confocal reflectance micrographs showing collagen fiber networks within the alginate gel. *p < 0.05; **p < 0.01; ***p < 0.001; ****p < 0.0001, not significant = ns.

The microenvironment of breast tumors is highly heterogeneous, exhibiting significant spatiotemporal variations in the physical and biochemical properties of the extracellular matrix (ECM) [1]. The progression of breast cancer involves increased collagen deposition, particularly fibrillar collagen I, and enhanced collagen fiber alignment within the ECM [2-4] (Fig. 1A), resulting in a tumor microenvironment that is significantly stiffer than healthy breast tissue. The increased collagen density and stiffness serve as important diagnostic and prognostic markers of disease [3,5,6], which can be assessed through manual palpation and radiographic imaging [7]. Additionally, greater collagen fiber alignment is correlated with poor patient survival rates [8]. Growing evidence also suggests that breast tumors exhibit viscoelastic properties, meaning they relax stress rapidly in response to applied deformation [9-11]. Furthermore, differences in viscoelasticity have been shown to discriminate between malignant and benign breast tumors [12,13]. While in vivo studies have documented simultaneous changes in multiple mechanical and biochemical ECM properties, how cells integrate these diverse cues to regulate malignant traits remains unclear.

In vitro models have been widely employed to investigate the influence of individual tumor microenvironmental cues on cell cycle progression [9], invasion [14], differentiation [15], and metabolism [16]. Hydrogel-based platforms mimicking tumor stiffness have been shown to promote invasion in both breast cancer and non-malignant breast epithelial cells [6,17]. Similarly, matrices with fast stress relaxation have been found to induce invasive cell morphologies, enhance migration, and activate cancer-associated signaling pathways in breast epithelial cells [10,18-21]. Additionally, high collagen density and fiber alignment have also been shown to modulate invasion and migration in both normal and malignant cell lines [22-25]. Collagen I can also facilitate invasion through *β*1 integrin clustering [6] and activation of ERK1/2 signaling, leading to increased epithelial to mesenchymal transition (EMT) [26,27]. Further, collagen I promotes focal adhesion formation and loss of cadherins, resulting in downstream activation of Rac1 and PI3K signaling [28,29]. Cells sense these ECM mechanical and biochemical cues through integrin-mediated focal adhesions and mechanosensitive ion-channels, which transduce signals via the actomyosin cytoskeleton to the nucleus, ultimately driving changes in gene expression [30-32]. Gene expression is regulated, in part, by transcriptional regulators, such as YAP/TAZ and MRTF, that are mechanically responsive [19,33,34]. However, compared to ECM stiffness, our knowledge of how cells sense and respond to ECM viscoelasticity remains severely lacking. Furthermore, while it is well established that individual tumor-mimicking ECM cues can enhance malignant traits in cancer cells, how cells generate a cumulative response to multiple cues in complex microenvironments remains poorly understood, primarily because decoupling these cues remains a challenge.

To address this challenge and better recapitulate the tumor ECM properties, we incorporated fibrillar collagen I into alginate hydrogels, thereby enabling independent tuning of stiffness, stress relaxation, and collagen density. We encapsulated mammary epithelial and breast cancer cells, breast cancer spheroids, and murine mammary organoids within these 3D matrices to study the effect of multiple tumor-mimicking ECM cues on cell invasion, migration, and gene expression. We reveal a novel signaling axis for the transcription factor Sp1 that regulates invasion in response to high stiffness and fast stress relaxation.

## 2. Results

We aimed to create a platform to tune ECM stiffness, stress relaxation, and collagen I density independently to determine how mammary epithelial and breast cancer cells respond when each property is varied alone or collectively. Using an alginate-collagen I interpenetrating network hydrogel, we developed two conditions for each property to mimic healthy or diseased extracellular matrices (Fig. 1B and C): soft or stiff (100 Pa vs. 2–4 kPa) [2], slow or fast stress relaxing (≈ 100 s vs. ≈ 1000 s stress-relaxation half-time) [11,35], and low or high collagen density (0.5 mg/ml vs. 2 mg/ml) [36-38]. The elastic modulus was varied in these matrices by changing the calcium ion concentration used to ionically crosslink the alginate chains. Stress relaxation time was varied by using alginates of different molecular weights [39]. Collagen density was controlled by incorporating varying amounts of type I collagen into the alginate matrices. Using shear rheology, we confirmed that stiffness could be tuned independently of stress relaxation half-time and collagen density (Fig. 1D). Similarly, stress relaxation half-times could be varied from thousands of seconds in slow relaxing matrices to hundreds of seconds in fast relaxing matrices, independent of the stiffness and collagen density (Fig. 1E and F). Further, upon varying collagen density in these matrices, we saw no significant differences in collagen fiber length, width, or orientation (Fig. 1G, Supp. Fig. 1A-C), consistent with findings from a recent study [40]. Thus, by varying these three properties over two distinct values, we generated eight unique matrix conditions that can be represented as vertices on a 3D state-space of the tumor microenvironment (Fig. 1C).

**Fig. 2.**
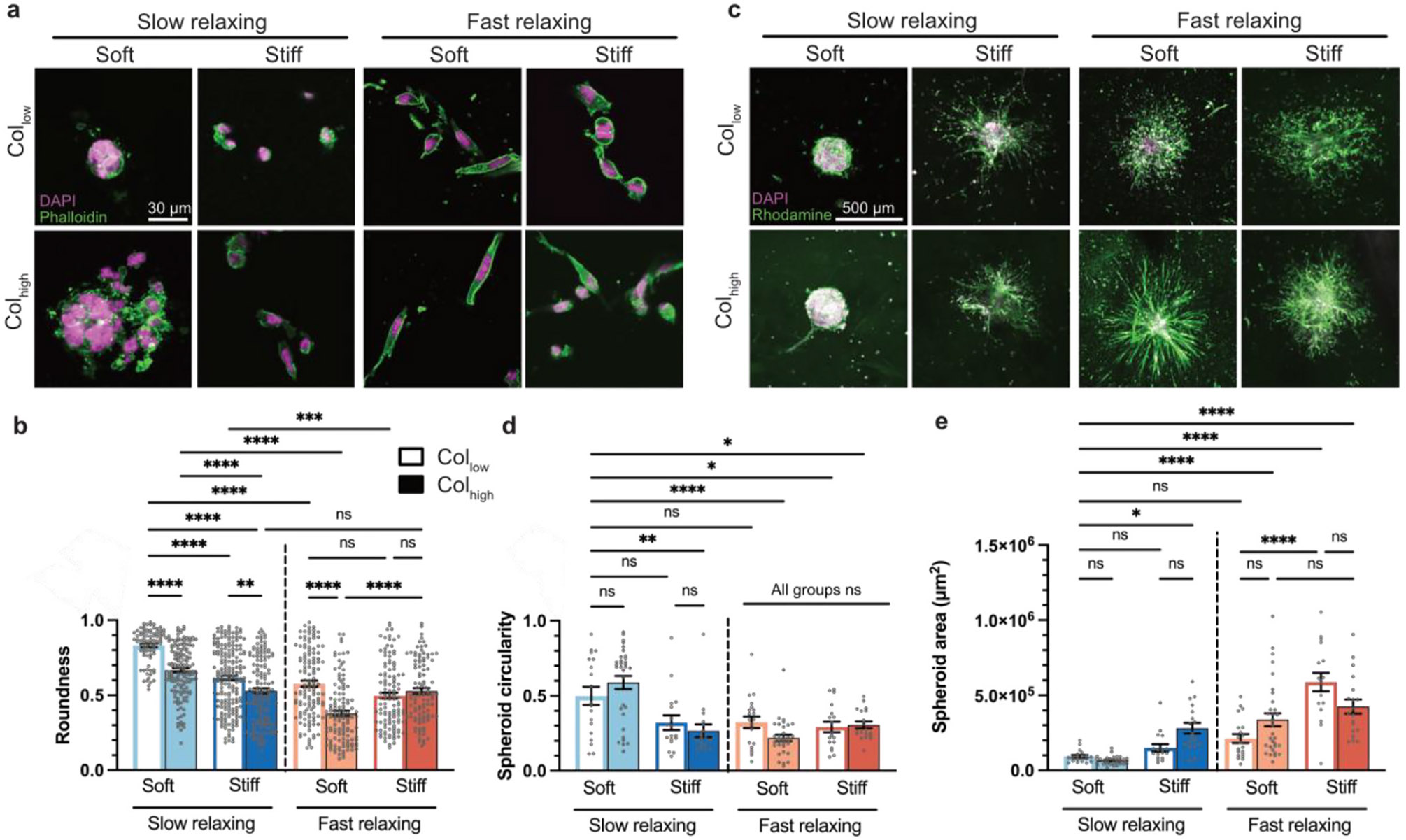
MDA-MB-231 cells and spheroids show invasive morphologies in the presence of individual and multiple tumor-mimicking ECM cues. **a,** Single cells were encapsulated in all 8 alginate-collagen matrix conditions and allowed to grow into clusters over a period of 7 days. **b,** Quantification of cell cluster roundness showed Soft-Slow-Col_low_ condition led to clusters with the highest roundness. High stiffness, fast stress-relaxation, high collagen, or combinations of these cues led to a significant decrease in roundness (n ≥ 20 cells per replicate, with 3 independent replicates; mean ± s. e.m.; Welch ANOVA test with post-hoc Dunnett’s multiple comparison test) **c,** Spheroids stained with DAPI and Octadecyl Rhodamine-B dye (R-18) were encapsulated and allowed to invade the matrix over a 3-day period. **d, e,** Quantification of spheroid circularity and area. Highest circularity and smallest area were observed in the Soft-Slow-Col_low_ and Soft-Slow-Col_high_ conditions. Compared to Soft-Slow-Col_low_, the presence of two or all three out of high stiffness, fast relaxation, or high collagen led to significant decrease in circularity and increase in area (n ≥ 5 spheroids per replicate, with 3 independent replicates; mean ± s. e.m.; ANOVA followed by post-hoc Sidak’s multiple comparison test was performed). *p < 0.05; **p < 0.01; ***p < 0.001; ****p < 0.0001, not significant = ns.

Morphology at the single-cell level has been previously shown to correlate with gene expression patterns and tumorigenicity [41-43]. To this end, metastatic breast adenocarcinoma cells (MDA-MB-231) were encapsulated as single cells within these eight matrix conditions and morphologically characterized after 7 days of culture. We measured roundness as the inverse of aspect ratio for individual cells or cell clusters if cells were in contact with neighboring cells. Cells in the Soft-Slow-Col_low_ matrix condition showed highly rounded morphologies and the greatest roundness value among all matrix conditions (Fig. 2A and B). Compared to the Soft-Slow-Col_low_ condition, the presence of high stiffness, fast stress relaxation, or high collagen density individually led to a statistically significant reduction in cell or cluster roundness (≈ 26 %, 31 %, and 20 %, respectively). This is in agreement with previous studies showing that epithelial cells adopted invasive morphologies in response to either of these cues [6,19,35,44,45]. We observed even larger reductions in cluster roundness with respect to the Soft-Slow-Col_low_ condition when two or all three cues were presented collectively at tumor-mimicking levels compared to just one. For example, the presence of both high stiffness and fast relaxation led to ≈ 40 % decrease in roundness. Thus, the combined effect of multiple tumor-mimicking ECM cues has a greater impact on cell morphology than any one individual cue, demonstrating a synergistic interaction between ECM cues. Additionally, we observed instances where a tumor-mimicking cue did not significantly affect cluster roundness if another cue was already present. For instance, in Stiff-Fast- Col_low_ versus Stiff-Fast-Col_high_ matrices, the presence of high collagen density showed no significant decrease in roundness. Similarly, in the Stiff-Slow-Col_high_ versus

Stiff-Fast-Col_high_ matrices, fast stress relaxation also led to no significant change in cluster roundness. These findings suggest that, in certain contexts, a single tumor-mimicking cue may not be sufficient to override the influence of other ECM cues in driving changes in cell morphology.

We also repeated this study in a tumorigenic but non-metastatic cell line (MCF-7), and a non-tumorigenic cell line (MCF-10A) to understand whether other mammary epithelial cell lines of different tumorigenicity show similar changes in cell morphology in response to multiple tumor-mimicking cues. In both these cell lines, we also observed a significant decrease in cell cluster roundness in response to one or multiple cues compared to the Soft-Slow-Col_low_ condition (Supp. Fig. 2A-D). Out of all three cues, MCF-10A cells showed the greatest decrease in roundness in response to high collagen (≈27 %) in comparison to fast relaxation (≈17 %) or high stiffness (≈14 %). In contrast, MDA-MB-231 cells showed the most pronounced reduction in roundness in response to fast stress relaxation. Furthermore, MCF-10A cells showed a distinct sensitivity to collagen concentration, with significant differences in roundness between the Stiff-Fast-Col_low_ and Stiff-Fast-Col_high_ groups, an effect not observed in MDA-MB-231 cells. The pre-malignant breast epithelial cell line MCF-10AT exhibited a significant reduction in roundness in matrices with high stiffness and fast stress relaxation (Supp. Fig. 2E and F). However, increased collagen density was not sufficient to induce significant changes in roundness. These findings suggest that different cell lines exhibit varying sensitivities towards tumor-mimicking ECM cues.

To investigate whether changes in morphology from encapsulated single cells were indicative of an invasive phenotype, we encapsulated MDA-MB-231 spheroids in alginate-collagen matrices and analyzed their invasiveness after 3 days of culture. Spheroid invasion was evaluated using circularity, calculated as the ratio of spheroid area to the square of its perimeter, which captures shape irregularities during invasion. We saw that MDA-MB-231 spheroids in the Soft-Slow-Col_low_ and Soft-Slow-Col_high_ matrix conditions were not invasive and showed the highest circularity and smallest invasion area (Fig. 2C and D). Notably, compared to the Soft-Slow-Col_low_ condition, the presence of high stiffness, fast relaxation, or high collagen density individually led to no significant changes in spheroid circularity or area. (Fig. 2D and E). The presence of two or all three tumor-mimicking cues, however, led to a significant decrease in circularity and an increase in spheroid area (Fig. 2C,D,E). Thus, the presence of multiple tumor-mimicking ECM cues has a greater impact on the cell-invasive phenotype than individual cues.

MCF-10A spheroids also showed a significant decrease in circularity and an increase in area in response to multiple cues (Supp. Fig. 3A-C). These spheroids in Soft-Slow-Col_low_ matrices showed a significantly larger decrease in circularity in the presence of high collagen density (≈30 %) compared to MDA-MB-231 spheroids (≈2 %) (Fig. 2D, Supp. Fig. 3A and B). This highlights their greater sensitivity towards high collagen density, similar to the trends seen in single cell encapsulation studies (Supp. Fig. 2D).

We also encapsulated murine mammary organoids in all 8 matrix conditions and observed changes in their morphology over a period of 2 weeks. Consistent with our single cell and spheroid encapsulation studies, we observed that the organoids remained round and did not invade the matrix in the Soft-Slow-Col_low_ condition (Supp. Fig. 3D). While organoids were less rounded and showed an invasive morphology in all stiff conditions, in the Soft-Slow-Col_high_ condition, the organoids elongated and branched similar to events observed during mammary morphogenesis in organoid cultures [46,47]. This suggests that mammary organoids can undergo invasion or morphogenesis depending on the presence of specific ECM cues and their combinations in their environment.

Increased collagen fiber alignment is a prognostic signature of poor survival in breast cancer patients [8]. We therefore determined if cells were also aligning the collagen fibers during invasion. Collagen fiber alignment was measured with respect to the cell boundary in all 8 matrix conditions after 7 days of culture. We quantified aligned collagen fibers as the proportion of total fibers oriented at an angle greater than 70° relative to the cell boundary. We observed that MDA-MB-231 cells showed the smallest fraction of aligned fibers in the Soft-Slow-Col_low_ condition (Supp. Fig. 4A and B). All other matrix conditions showed a significantly higher fraction of aligned fibers. MCF-10A cells also showed a significantly higher percentage of aligned fibers compared to the Soft-Slow-Col_low_ group, except in the Stiff-Slow-Col_high_ condition (Supp. Fig. 4C). This observed reduction in fiber alignment in our high collagen matrix condition is in line with previous studies showing decreased collagen fiber orientation and cell alignment in high collagen matrices [48,49].

**Fig. 3.**
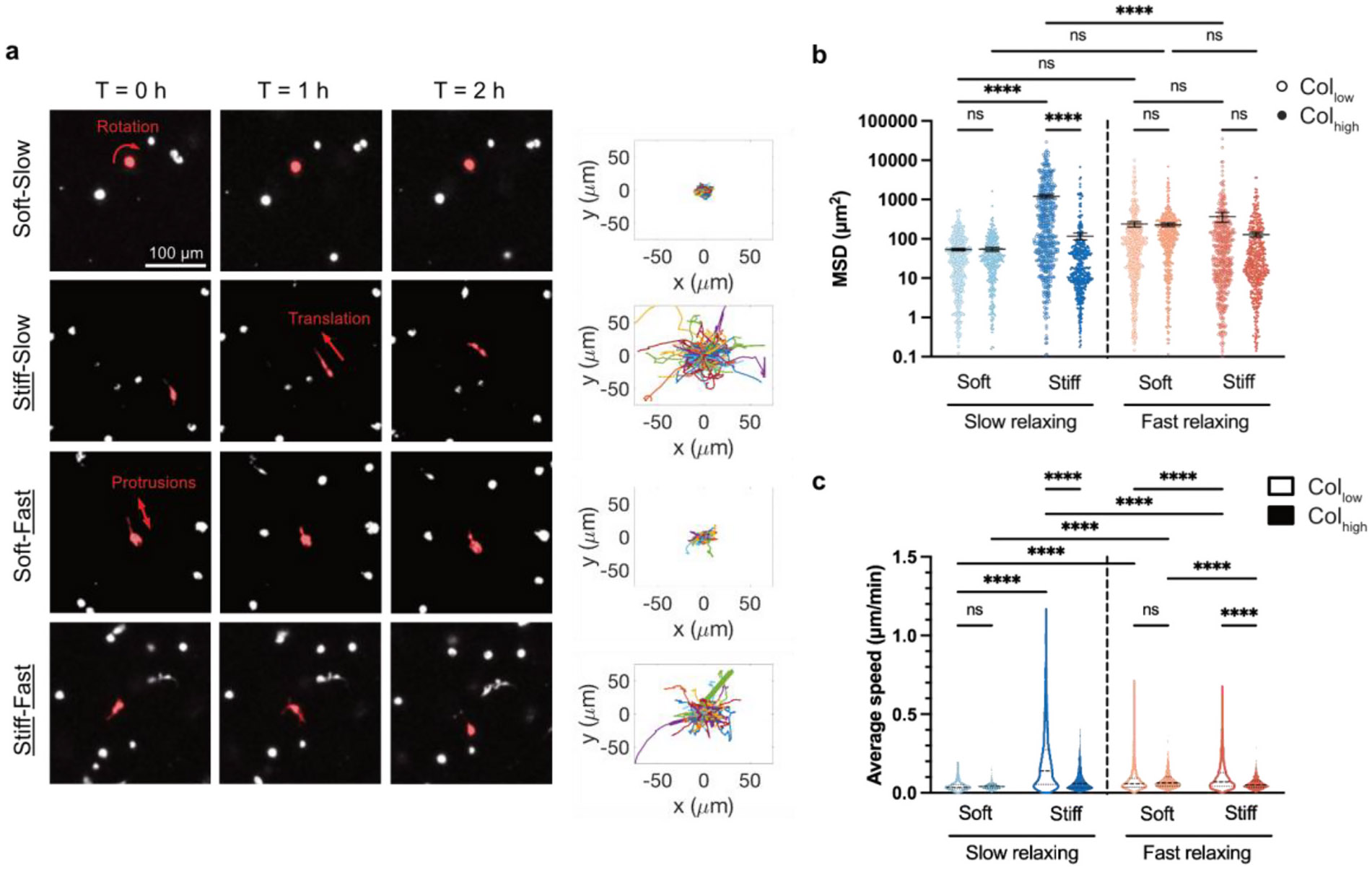
Matrix stiffness drives the largest increases in cell migration compared to other tumor-mimicking ECM cues. **a,** Live-cell imaging of MDA-MB-231 cells in Col_low_ matrices that were Soft-Slow, Stiff-Slow, Soft-Fast, or Stiff-Fast. The centroid of single cells was tracked and their migration trajectory projection in the X–Y plane is plotted. **b,** Mean squared displacement (MSD) and **c,** average speed were measured across all 8 matrix conditions. The greatest increase in MSD and average speed was observed in the Slow-Stiff-Col_low_ condition (n ≥ 100 cells per replicate, with 3 independent replicates; mean ± s. e.m.; ANOVA followed by post-hoc Tukey’s multiple comparison test was performed. *p < 0.05; **p < 0.01; ***p < 0.001; ****p < 0.0001, not significant = ns).

We next investigated the extent to which stiffness, stress relaxation, and collagen density impact the migration of MDA-MB-231 metastatic breast cancer cells. Using time-lapse confocal microscopy, we tracked cell positions over 16 h and found that cells in Soft-Slow-Col_low_ and Soft-Slow-Col_high_ conditions showed the lowest mean squared displacement (MSD) and average speed. Compared to the Soft-Slow-Col_low_ matrix condition, the presence of high stiffness led to the highest increase in MSD and average speed (Fig. 3A-C). While fast stress relaxation led to a significant increase in average speed but not MSD, high collagen led to no significant changes in either MSD or average speed. Cells in Soft-Slow-Col_low_ and Soft-Slow-Col_high_ conditions showed rotation around their centroid but no translation (Supp. Video 1,5), in line with previous studies [50,51]. Notably, fast stress relaxation promoted cellular protrusions that dynamically extended and retracted over time (Fig. 3A-Supp. Video 3), as previously reported [10,18]. In many cases, these dynamic protrusions did not lead to persistent migration, as measured by translation of the centroid of the cell, and thus are not accounted for in our MSD and speed metrics. Further, in the collective presence of high stiffness and fast stress relaxation, cells showed both increased stiffness-driven enhanced migration (Fig. 3A), as well as fast relaxation-driven formation of dynamic protrusions (Supp. Video 4,8). In Stiff-Slow-Col_low_ versus Stiff-Fast-Col_low_ conditions, the presence of fast stress relaxation induced a significant reduction in both MSD and speed (≈70 % MSD and ≈87 % average speed) (Fig. 3B-D). Similarly, compared to the Stiff-Slow-Col_low_ condition, the presence of high collagen density also led to a large and significant decrease in both MSD (≈936 %) and speed (≈107 %). This was also true in fast relaxing matrices, where, compared to the Stiff-Fast-Col_low_ condition, the presence of high collagen density also led to a significant decrease (≈57 %) in average speed and a decrease in MSD (≈184 %), though that change was not statistically significant. This observed reduction in cell migration in response to high collagen density is in line with previous studies [52]. In summary, we observe that the presence of multiple tumor-mimicking cues can lead to cell populations that display diverse phenotypic behaviors. Our results also depict that in complex microenvironments, cell migration in response to a specific ECM cue can be strongly influenced by the concurrent presence of other ECM cues.

**Fig. 4.**
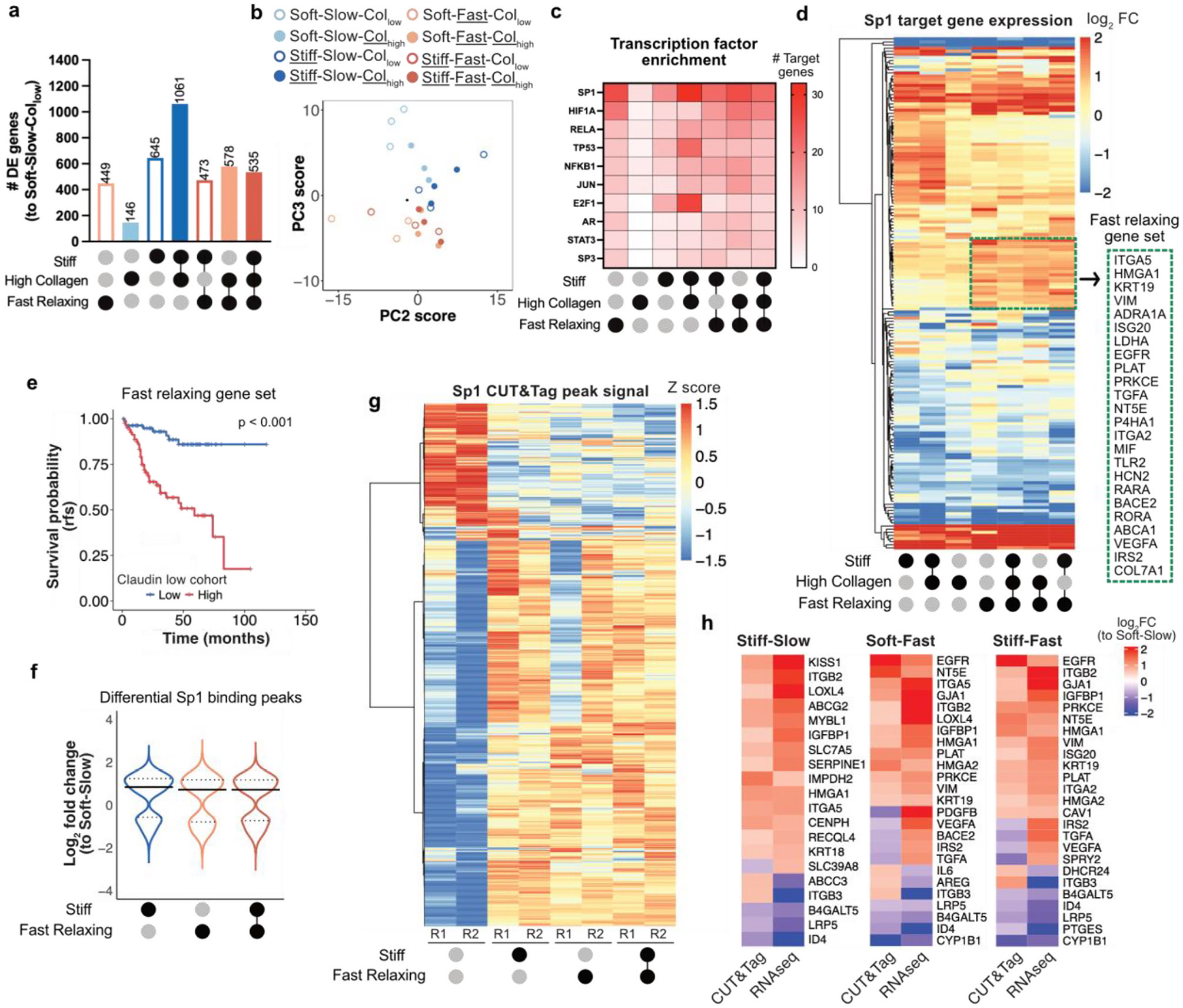
Tumor-mimicking ECM cues are associated with enrichment of Sp1 target genes and increased Sp1 binding. **a,** The number of differentially expressed genes in MDA-MB-231 cells was determined for all matrix conditions with respect to the Soft-Slow-Col_low_ condition. Stiff-Slow (Col_low_ and Col_high_) conditions showed the highest differential gene enrichment (n = 3 independent replicates per condition; abs log_2_ fold change >1, p < 0.05). **b,** PCA plot of RNA-seq data shows that fast relaxing groups cluster separately from the slow relaxing groups. **c,** Heat map showing the top transcription factors enriched using TRRUST database for each matrix condition. Sp1 target genes were highly enriched in presence of high stiffness and fast stress relaxation. **d,** Heatmap showing log_2_ fold change in expression for Sp1 target genes (obtained from TRRUST database) in all conditions compared to the Soft-Slow-Col_low_ condition. The gene set enclosed in the dashed box was upregulated in all fast relaxing conditions. **e,** Recurrence-free survival probability was plotted for n = 162 patients with the claudin-low breast cancer subtype using the fast relaxing gene set identified in panel d. High expression, defined by a median split, was associated with significantly reduced survival probability. Log rank p value is reported. **f,** Log_2_ fold change for CUT&Tag differential Sp1 binding peaks with respect to the Soft-Slow condition (n = 2 replicates per condition; p < 0.05). The median log_2_ fold change values were positive in matrices of high stiffness as well as fast stress relaxation, indicating increased Sp1 binding. Dotted lines represent the upper and lower quartiles. All conditions are Col_low_. **g,** Heatmap showing Z score-normalized CUT&Tag signal (counts per million) for all Sp1 binding sites. High stiffness and fast relaxation exhibit an enriched cluster of binding peaks compared to the Soft-Slow condition. All conditions are Col_low_ (n = 2 replicates, R1 and R2 per condition). **h,** Heatmaps showing log_2_ fold change of genes with differential Sp1 binding in MDA-MB-231 cells with respect to Soft-Slow condition and the corresponding log_2_ fold change in gene expression. Binding peak signal and gene expression with an absolute log_2_ fold change >0.5 were considered).

To investigate how gene expression is influenced by stiffness, stress relaxation, and collagen density, we performed RNA sequencing on cells cultured for 7 days in all eight matrix conditions. Differential gene expression analysis was conducted by comparing each matrix condition to the Soft-Slow-Col_low_ matrix as the control. For MDA-MB-231 cells, the largest number of differentially expressed genes were observed in the Slow-Stiff groups (Fig. 4A), while for MCF-10A cells, high collagen groups led to the most differentially expressed genes (Supp. Fig. 5A). Performing principal components analysis (PCA) on all 8 matrix conditions revealed that with MDA-MB-231 cells, fast relaxing matrix groups clustered separate from the slow relaxing groups (Fig. 4B). However, with MCF-10A cells, the high collagen groups were clustered together, consistent with their greater collagen sensitivity (Supp. Fig. 5B). In summary, these findings suggest that tumor-mimicking ECM cues perturb the transcriptome in a cell line-specific manner.

We next performed Gene Set Enrichment Analysis (GSEA) against the oncogenic signature gene set (C6) in the Human Molecular Signatures Database (MSigDB) [53,54]. Our analysis revealed that KRAS signaling, associated with disease progression in multiple solid cancers [55], was enriched in both MDA-MB-231 and MCF-10A cells in all conditions marked by high stiffness, fast relaxation, or high collagen, as well as combinations of these cues (Supp. Fig. 5C and D). This shows that our tumor-mimicking cue parameters lead to enrichment of multiple cancer-associated signaling pathways implicated in breast cancer.

We investigated transcription factor enrichment across various matrix conditions relative to the Soft-Slow-Col_low_ control using the TRRUST database [56]. Several well-known transcription factors associated with breast cancer were enriched (Fig. 4C, Supp. Fig. 5E). Among these, we found that Sp1, whose expression is inversely correlated with patient survival in multiple cancers [57], showed a high enrichment of target genes in both MDA-MB-231 and MCF-10A cells in response to high stiffness, fast relaxation, high collagen density, as well as the combination of these cues. This strong enrichment of Sp1 target genes aligns with prior studies demonstrating its role in mediating cellular response to matrix stiffness and direct mechanical force application [17,58], further emphasizing its implications in mechanoresponsive pathways. Notably, hierarchical clustering of log_2_ fold changes in gene expression, relative to the Soft-Slow-Col_low_ control, revealed that in MDA-MB-231 cells, a subset of Sp1 target genes was upregulated in all fast relaxing groups compared to the slow relaxing groups (Fig. 4D). Since MDA-MB-231 cells are considered as representative of claudin-low breast cancer [59], we compared this upregulated gene set enriched in fast relaxing conditions against claudin-low subtype breast cancer patients [60]. Interestingly, patients with high expression of this set of genes had significantly lower recurrence-free survival probability (Fig. 4E).

To determine whether the altered expression of Sp1 target genes in response to ECM mechanical cues correlated with differential Sp1 binding to genomic loci, we performed CUT&Tag sequencing [61] on MDA-MB-231 cells encapsulated in the Soft-Slow, Stiff-Slow, Soft-Fast, and Stiff-Fast groups, maintaining a low collagen density. CUT&Tag is a sequencing method analogous to ChIP-seq, that instead of immunoprecipitation, uses the Tn5 transposase to simultaneously cleave and tag DNA at antibody-bound chromatin, enabling a higher-resolution mapping with lower cell-input requirement [61]. We found a positive median log_2_ fold change in Sp1 binding peak signal in stiff as well as fast relaxing conditions (Fig. 4F), indicating enhanced Sp1 binding. Hierarchical clustering of all Sp1 binding peaks also showed that a large subset of peaks was more enriched in matrices with high stiffness or fast stress relaxation compared to the Soft-Slow group (Fig. 4G). Representative integrative genomics viewer (IGV) plots of Sp1 binding at the integrin ITGB2 and ITGA5 loci provide specific, illustrative examples (Supp. Fig. 5F and G). Further, we identified a subset of genes that exhibited both increased Sp1 binding and elevated gene expression, with a log_2_ fold change value greater than 0.5 relative to the Soft-Slow condition (Fig. 4H). Notably, higher expression of these gene sets, enriched in the fast relaxing groups (Soft-Fast and Stiff-Fast), was associated with a significant decrease in survival among the claudin-low subtype patients (Supp. Fig. 5H). Thus, our findings demonstrate that tumor-mimicking mechanical cues are associated with both increased Sp1 binding at genomic loci and enrichment of Sp1 target gene expression.

**Fig. 5.**
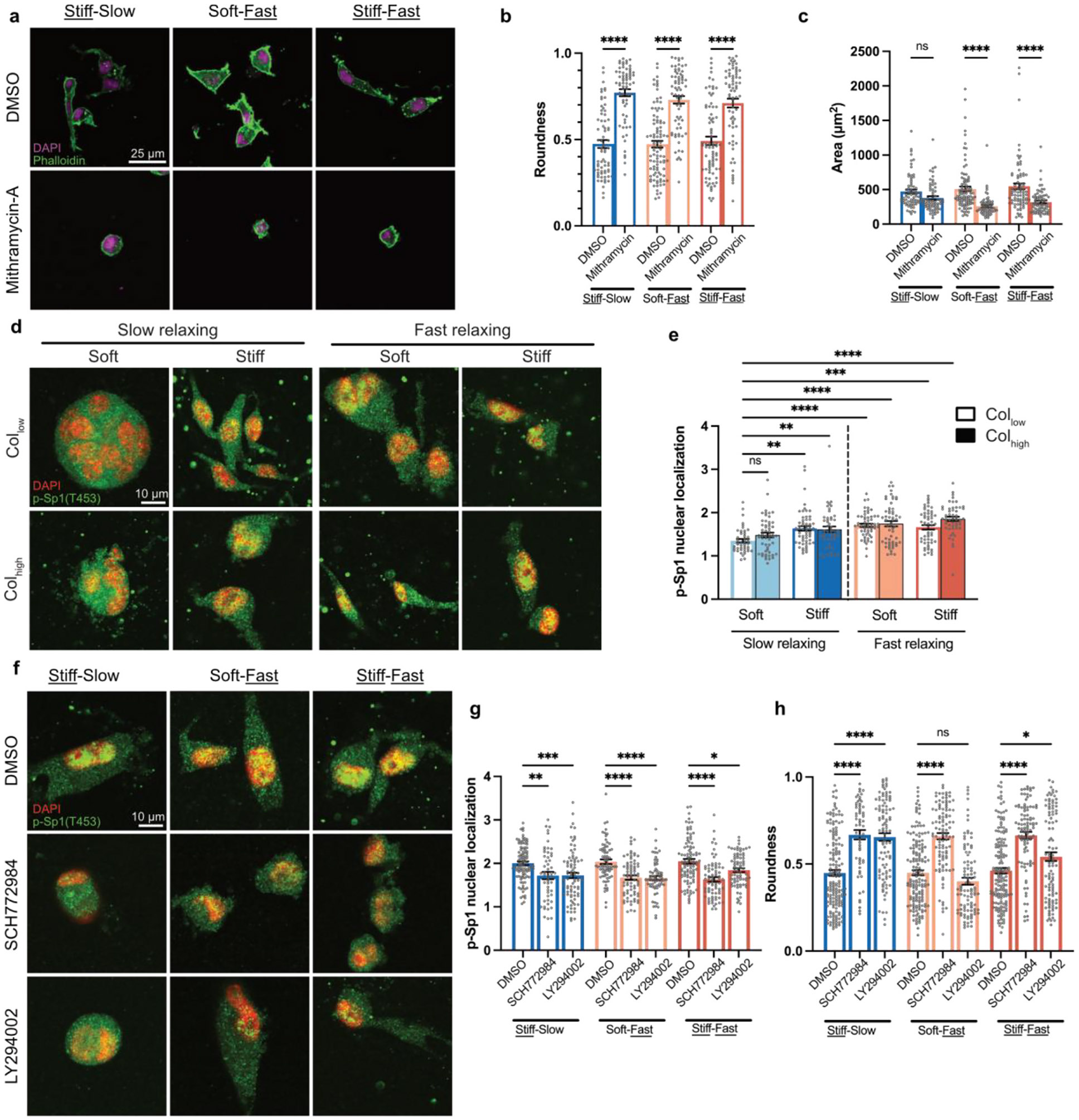
Sp1 induces the invasive phenotype in response to multiple tumor-mimicking ECM cues. **a,** Inhibiting Sp1 activity using mithramycin-A led to a significant decrease in cell MDA-MB-231 cluster roundness in Stiff-Slow, Soft-Fast, as well as Stiff-Fast matrix conditions. **b, c,** Quantification of the change in roundness and cluster area upon Sp1 inhibition (n ≥ 15 cells per replicate, with 3 independent replicates; ANOVA followed by Sidak’s multiple comparison test). **d,** Immunofluorescence imaging of phospho-Sp1(T453) and **e,** quantification of the nuclear to cytoplasmic ratio of mean intensity levels shows a significant increase in most matrix conditions compared to the Soft-Slow-Col_low_ condition (n ≥ 15 cells over 3 independent replicates; mean ± s. e.m; ANOVA followed by Dunnett’s multiple comparison test). **f,** Confocal immunofluorescence imaging of cells treated with ERK1/2 inhibitor (SCH772984) and PI3K inhibitor (LY294002). All conditions have low collagen density. **g,** Both ERK1/2 and PI3K inhibition led to a significant decrease in nuclear localization of phospho-Sp1(T453) in Stiff-Slow, Soft-Fast, as well as Stiff-Fast matrix conditions. **h,** Cluster roundness was quantified for phalloidin stained cells. While ERK1/2 inhibition led to significant decrease in all conditions, PI3K inhibition had a significant effect only in stiff conditions. For both **g, h,** n ≥ 15 cells per replicate, with 3 independent replicates; mean ± s. e.m.; ANOVA followed by Sidak’s multiple comparison test was performed. *p < 0.05; **p < 0.01; ***p < 0.001; ****p < 0.0001, not significant = ns).

Next, we sought to determine the role of Sp1 in regulating the malignant phenotype driven by matrix mechanics. Although Sp1 is known to play a role in cancer progression [57], its role as a mechanotransducer is understudied. To this end, we used mithramycin-A, a well-established small molecule inhibitor of Sp1 [62,63] to treat MDA-MB-231 and MCF-10A cells encapsulated in matrices with high stiffness, fast relaxation, or the presence of both these mechanical cues (Stiff-Slow, Soft-Fast, and Stiff-Fast). Sp1 inhibition led to a significant increase in cell roundness for both cell types in all three treated groups compared to the DMSO vehicle controls (Fig. 5A and B, Supp. Fig. 6A and B). While MDA-MB-231 cells showed a significant decrease in cluster area in fast relaxing conditions only (Fig. 5C), MCF-10A cells showed a significant decrease in area in all three treatment conditions (Supp. Fig. 6C).

Increased phosphorylation of Sp1 at Thr-453 has been previously associated with the formation of invasive cell clusters in stiff alginate-Matrigel hydrogels [17]. Further, Sp1 phosphorylation has been shown to increase its transcriptional activity [64-66]. To this end, we investigated whether there are changes in levels of Sp1 phosphorylation (T453) in response to tumor-mimicking ECM cues that drive the invasive phenotype. Notably, in MDA-MB-231 cells, we observed that compared to the Soft-Slow-Col_low_ condition, there was a significant increase in levels of nuclear-to-cytoplasmic ratio of phospho-Sp1 (T453) in all stiff and fast relaxing groups, correlating with the occurrence of invasive morphologies (Fig. 5D and E). MCF-10A cells, however, showed high levels of phospho-Sp1 nuclear localization even in the Soft-Slow-Col_low_ control condition, and no significant changes in response to most ECM cues, except in the Soft-Fast-Col_low_ and Stiff-Slow-Col_high_ conditions (Supp. Fig. 6D and E). MCF-10AT cells also showed increased localization of nuclear phospho-Sp1 in stiff, fast relaxing, as well as high collagen groups (Supp. Fig. 6F and G). To test whether the presence of collagen I in the matrix contributes to elevated baseline levels of phospho-Sp1 nuclear localization, we also encapsulated MCF-10A cells in alginate-reconstituted basement membrane (rBM) matrices with similar stiffness and stress relaxation to the collagen-based matrices used throughout this study (Supp. Fig. 7A-C). Stiff and fast relaxing matrices induced invasive morphologies (Supp. Fig. 7D), characterized by a significant decrease in roundness (Supp. Fig. 7E) and increased nuclear phospho-Sp1 localization (Supp. Fig. 7G), relative to the.

Soft-Slow alginate-rBM control. Moreover, significantly higher phospho-Sp1 nuclear localization was observed in the Soft-Slow alginate-collagen groups (both Col_low_ and Col_high_) compared to alginate-rBM (Supp. Fig. 7H). In summary, our findings demonstrate that both matrix mechanics as well as the type of ligand can contribute to the nuclear localization of phospho-Sp1.

Both ERK1/2 and PI3K are known to phosphorylate Sp1 at Thr-453 and in various cell types, including breast cancer cells [67-70]. Interestingly, it has been shown that Sp1 is differentially phosphorylated in response to 3D matrix stiffness, as well as upon direct mechanical force application [17,58]. We therefore asked whether phosphorylation of Sp1 at Thr-453 by ERK1/2 or PI3K is critical for driving invasion in response to tumor-mimicking ECM cues. Both ERK1/2 inhibition with SCH77298 and PI3K inhibition with LY294002 led to a significant decrease in phospho-Sp1 nuclear localization in MDA-MB-231 and MCF-10A cells in response to high stiffness, fast relaxation, or the presence of both these mechanical cues (Fig. 5F and G, Supp. Fig. 8A and B). MDA-MB-231 cells plated on 2D glass also exhibited reduced phospho-Sp1 nuclear localization upon ERK1/2 or PI3K inhibition, with an even greater reduction following their combined inhibition (Supp. Fig. 8D and E). In MCF-10A cells, ERK1/2 or PI3K inhibition alone produced only a non-significant decrease, though the combined inhibition resulted in a significant reduction in nuclear phospho-Sp1 localization (Supp. Fig. 8D and F). Further, ERK1/2 inhibition also led to a significant increase in cluster roundness in all three mechanical conditions in both cell lines encapsulated in alginate-collagen matrices (Fig. 5H, Supp. Fig. 8C). While PI3K inhibition in MDA-MB-231 cells led to a significant increase in roundness in stiff matrices independent of stress relaxation (Stiff-Slow, Stiff-Fast), MCF-10A cells showed a significant increase in roundness in the slow relaxing (Stiff-Slow) condition only. Our results show that Sp1 mechanosignaling drives the malignant phenotype in response to specific tumor-like matrix mechanical properties via phosphorylation at Thr453 by ERK1/2 and PI3K.

**Fig. 6.**
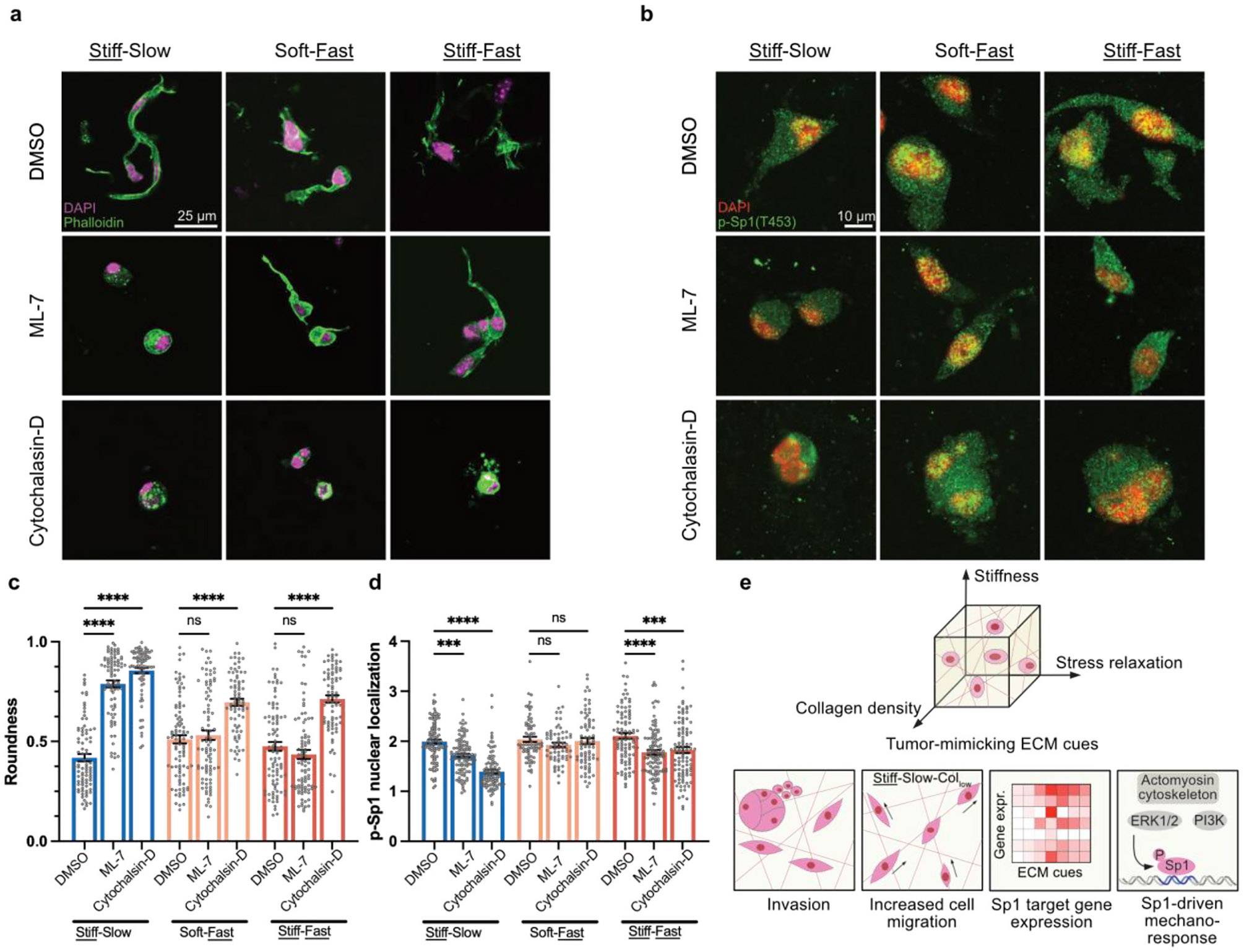
Actomyosin cytoskeleton regulates phospho-Sp1 nuclear localization in stiff matrices. **a, b** Myosin light chain kinase (MLCK) was inhibited via ML-7 and actin polymerization was inhibited via cytochalasin D in MDA-MB-231 cells encapsulated in Stiff-Slow, Soft-Fast, as well as Stiff-Fast matrix conditions (Col_low_). Confocal imaging was performed on phalloidin, DAPI, and phospho-Sp1(T453) stained MDA-MB231 cells. **c,** Inhibition of MLCK led to a significant increase in cluster roundness only in Stiff-Slow relaxing condition, and inhibition of actin polymerization led to a significant increase in roundness in all 3 conditions. **d,** MLCK and actin polymerization inhibition led to a significant decrease in nuclear localization levels of phospho-Sp1 only in stiff matrices (Stiff-Slow and Stiff-Fast). **e,** Summary schematic showing that in complex environments with multiple tumor-mimicking ECM cues such as high stiffness, altered stress relaxation, and high collagen density, cells undergo changes in their morphological and migratory phenotype. Multiple ECM cues can also act synergistically at the transcriptional and signaling pathway level. We observed enrichment of Sp1 target genes in response to multiple ECM cues. Sp1 phosphorylation is regulated via the actomyosin machinery and ERK1/2 and PI3K kinases, which drives the downstream malignant traits. For both **c, d,** n ≥ 15 cells per replicate, with 3 independent replicates; mean ± s. e.m.; ANOVA followed by Sidak’s multiple comparison test. *p < 0.05; **p < 0.01; ***p < 0.001; ****p < 0.0001, not significant = ns.

The actin cytoskeleton has been previously shown to affect cell response to ECM mechanical cues either via direct force transmission to the nucleus or through downstream signaling cascades [30,71]. We therefore asked whether actomyosin contractility also regulates the occurrence of invasive morphologies in breast cancer cells via Sp1 signaling. Since contractility in non-muscle cells is driven by phosphorylation of myosin via myosin light chain kinase (MLCK) [72-74], we used an MLCK inhibitor, ML-7, to disrupt cell contractility. In ML-7 treated groups, both MDA-MB-231 and MCF-10A cells showed a significant increase in roundness, but only in the Stiff-Slow matrix condition, with no significant effect observed in fast relaxing matrices (Soft-Fast, Stiff-Fast) (Fig. 6A-C, Supp. Fig. 9A and B). Since it has been previously demonstrated that cells respond to fast relaxing environments by forming actin-rich protrusions [10,18,19,75], we disrupted actin network polymerization using Cytochalasin-D. Compared to DMSO-treated controls, both MDA-MB-231 and MCF-10A cells treated with Cytochalasin-D showed a significant increase in roundness in the presence of high stiffness, as well as fast stress relaxation (Stiff-Slow, Soft-Fast, Stiff-Fast). Notably, MDA-MB-231 cells treated with either ML-7 or Cytochalasin-D showed a significant decrease in nuclear localization of phospho-Sp1 only in stiff matrices (Stiff-Slow and Stiff-Fast), but not in the Soft-Fast matrix (Fig. 6A-D). MCF-10A cells exhibited a unique response where ML-7 treatment led to reduced phospho-Sp1 nuclear localization only in the slow relaxing, stiff matrix condition (Stiff-Slow), however, Cytochalasin-D treatment led to reduced nuclear localization across all three matrix conditions, in line with their increased roundness (Supp. Fig. 9A-C). Thus, actin polymerization regulates phospho-Sp1 nuclear localization in MCF-10A cells across stiff, as well as fast relaxing tumor-mimicking mechanical conditions, whereas in MDA-MB-231 cells, this regulation is specific to stiff environments. In summary, we observed that induction of invasive cell morphologies occurs in a mechanical cue and cell-line-specific manner via myosin contractility and actin polymerization, which drives downstream changes in phospho-Sp1 nuclear localization.

## 3. Discussion

The tumor ECM presents a heterogeneous microenvironment where a complex interplay of mechanical and biochemical cues collectively governs cellular invasion within the tissue. Our reductionist approach, using alginate-collagen hydrogels with independently tunable properties, has allowed us to systematically vary stiffness, stress relaxation rates, and collagen density, providing a controlled platform to dissect their individual and combined effects. Using multiple cell lines, spheroids, and murine mammary organoids, we show that ECM physical and biochemical cue combinations can generate diverse phenotypes. We identified that a decrease in cell and cluster roundness is reinforced when multiple tumor-associated cues are present in the microenvironment surrounding the cells. However, at the transcriptomic level, the presence of multiple tumor-mimicking cues did not always yield a greater number of differentially expressed genes, suggesting possible contributions from post-translational modifications, amplification of existing pathways across multiple cues, and non-linear interactions. In addition, cellular heterogeneity may also mask some of these effects when assessed by bulk RNA-seq. Future studies investigating which ECM cues most critically drive invasive behavior across different cell lines could provide valuable insights for developing targeted therapeutic strategies in breast cancer treatment.

During cancer invasion and metastasis, the migration of tumor cells has been shown to be regulated by various physical cues within the tumor microenvironment [10,76,77]. In this study, we observed that matrix stiffness, stress relaxation, and collagen density together dictate cell migration. For instance, in soft matrices, MDA-MB-231 cells migrate with greater speed in the presence of fast stress relaxation, however, in stiff matrices, this trend is reversed. These observations align with previous findings reporting enhanced migration of MCF-10A cells on soft, fibronectin-coated viscoelastic substrates but reduced migration on stiff substrates [78]. However, a recent report showed that MDA-MB-231 cells on Matrigel-coated 2D substrates demonstrated increased migration in response to fast stress relaxation on substrates with a Young’s modulus similar to our stiff matrices [18]. These differences might be attributed to differing modes of migration in 2D versus 3D and laminin/collagen IV ligands employed in their study, known to trigger distinct integrin-mediated signaling pathways [79]. Such variations underscore the importance of matrix composition, dimensionality, and ligand specificity in modulating cell migration behavior.

It is well established that breast tumors are stiffer than healthy breast tissue. In vitro experiments have shown that increased stiffness drives malignant traits and is correlated with poor patient survival in vivo [80,81]. While some recent studies have shown that breast tumors are viscoelastic, exhibiting rapid stress relaxation [9,11,12], there is relatively limited mechanical characterization of viscoelasticity in breast tissue and tumors, particularly with respect to alterations during disease progression. Thus, we chose stress relaxation times on the same order of magnitude as the reported values for breast tumor for our fast stress relaxation group and varied that by an order of magnitude for the slow stress relaxation group. Notably, we observed significant changes across several aspects of cellular phenotype, showing that breast cancer cells are responsive to this degree of matrix stress relaxation. Other studies have also employed hydrogels displaying stress relaxation times within this range to model breast cancer and changes in cell phenotype [9,18,82].

Although extensive research has focused on elucidating the pathways involved in mechanotransduction in response to varying elastic moduli during disease progression, how cells sense and respond to changes in ECM viscoelasticity remains less well understood. To our knowledge, our study is the first to implicate Sp1 signaling as a regulator of cellular response to matrix stress relaxation. Consistent with this role, we observed altered nuclear localization of phospho-Sp1 in response to matrix stiffness, stress relaxation, ligand density, as well as the ligand type. These findings suggest that the Sp1 signaling axis may be engaged by multiple tumor-mimicking ECM cues, although crosstalk with other pathways is also likely to play an important role in driving invasion. We further demonstrate that Sp1 exhibits increased genomic binding under both stiff and fast relaxing conditions. Notably, Sp1 target genes that were upregulated across fast relaxing conditions and showed a concordant increase in Sp1 binding were associated with poor patient survival, underscoring a potential role for Sp1-dependent gene regulation in promoting disease progression. High expression of Sp1 is known to correlate with poor prognosis and shorter survival times in multiple cancers, including breast cancer [57,83]. Single-cell transcriptomic analysis of patient-derived tumors has also identified Sp1 as a key regulator of metastasis across multiple cancer types [84]. Sp1 undergoes a large number of post-translational modifications that regulate its stability and transcriptional activity [57]. Previous work has shown that Sp1-HDAC3/8 signaling can drive the malignant phenotype via changes in chromatin accessibility in response to increased ECM stiffness in mammary epithelial cells [17]. Another study has previously reported that direct force application to fibroblasts via collagen-coated beads led to higher levels of phospho-Sp1 [58]. However, the role of Sp1 and its downstream modifications in response to matrix mechanical remodeling in breast cancer remains understudied. ERK1/2 acts as a downstream effector in Ras signaling that can shuttle into the nucleus [85,86] and phosphorylate multiple transcription factors such as Sp1 and HIF1A, c-Myc, STATs, Jun, and Fos [66,67,87]. PI3K has also been shown to phosphorylate Sp1 and regulate downstream gene expression [69,88]. We demonstrate that Sp1 phosphorylation through ERK1/2 and PI3K signaling pathways regulates cell invasion in response to matrix mechanics.

Cells sense ECM mechanical cues via integrin-mediated focal adhesions, which regulate downstream pathways such as MEK, ERK, and PI3K [89]. Sp1 has been previously shown to directly bind to multiple YAP1 promoter regions during pancreatic carcinogenesis [90]. Our RNA-seq results also showed enrichment of YAP-regulated genes in both stiff and fast relaxing matrices. Further, YAP has been shown to regulate KRAS and HRAS signaling pathways, leading to changes in pERK and pMEK levels [91]. This could point towards a possible crosstalk with Sp1 signaling in breast cancer. RhoA/ROCK signaling has also been shown to regulate ERK and PI3K signaling in a FAK-dependent manner [92,93]. Further, there is also evidence showing that both ERK and PI3K regulate Rac1 activity and downstream cytoskeletal remodeling [94,95]. All these studies suggest that Sp1 regulation may have substantial overlap with other well-established mechanosignaling pathways.

We also observed loss of the invasive morphologies, as well as downregulation in nuclear localization of phospho-Sp1 in both MDA-MB-231 and MCF-10A cells upon inhibiting actin-polymerization in stiff matrices. It is known that ERK can interact with the actomyosin cytoskeleton and favor actin polymerization [96]. Chemical and optogenetic induction of cell protrusions, which are characterized by increased actin polymerization, have also been shown to activate ERK signaling [97], while inhibition of actomyosin contractility can abrogate downstream ERK signaling [98]. These findings might also help explain changes in the nuclear localization of phospho-Sp1. Intriguingly, in MDA-MB-231 cells, we did not observe changes in phospho-Sp1 nuclear localization in Soft-Fast matrices. Future work is needed to determine the role of other cytoskeletal elements, or their crosstalk in regulating Sp1 signaling in these matrices.

In summary, we modeled key features of the breast tumor microenvironment, including increased stiffness, fast stress relaxation, and high collagen density, using alginate-collagen hydrogels that allowed us to independently tune these properties. Our findings demonstrate that these tumor-mimicking ECM cues can concurrently influence cell morphology, invasion, and migration, and that their effects can converge on shared regulators such as Sp1. We identified Sp1 and the nuclear localization of its phosphorylated form as a regulator of invasion in response to mechanical cues, with this nuclear localization regulated by ERK and PI3K signaling as well as by the actin cytoskeleton. Furthermore, Sp1 target genes were enriched in stiff, fast relaxing, and high collagen density groups. Importantly, upregulation of Sp1 target genes enriched in fast relaxing matrices, which also exhibited increased Sp1 binding, correlated with poor survival in claudin-low breast cancer patients. Together, these findings highlight the need to further investigate Sp1 mechanosignaling as a potential contributor to breast cancer progression and a candidate therapeutic target.

## 4. Methods

### 4.1. Preparing 3D alginate matrices

Pronova^®^ UP-VLVG (MW < 75 kDa) and LF20/40 (265 kDa) sodium alginates were used for producing fast and slow relaxing matrices, respectively. Calcium sulfate dihydrate (Sigma) was used to ionically crosslink the alginate chains. 0.5 mg/ml or 2 mg/ml rat tail collagen I (Advanced Biomatrix) was incorporated to form the alginate-collagen interpenetrating network (IPN) matrices. Briefly, the alginate solution in a Luer-lock syringe was mixed with collagen I and calcium sulfate solutions diluted in DMEM or DMEM/F12 in a second Luer-lock syringe. Collagen was neutralized using 1 M NaOH before mixing with the alginate solution. The syringes were then coupled using a Luer-lock, and the solution was mixed back and forth 20 times before depositing it into the well plates. For reconstituted basement membrane (rBM)-based matrices, growth factor-reduced Matrigel^®^ (Corning) was kept on ice and mixed with the alginate solution at the desired concentration. The solution was then allowed to gel for 2 h at 37 °C. The exact recipe for each matrix condition can be found in Supp. Table 1.

### 4.2. Single cell encapsulation and cell culture

MCF-7 and MDA-MB-231 cells were cultured in media with DMEM (4.5 g/L d-glucose, ThermoFisher Scientific), 10 % fetal bovine serum (ThermoFisher Scientific), and 1 % Penicillin/Streptomycin (ThermoFisher Scientific). Cells were used up to passage 40. Cells were encapsulated at 250,000 cells/ml and cultured in gels for 7 days. MCF-10A and MCF-10AT cells were cultured in media containing DMEM/F12 (ThermoFisher Scientific), 5 % horse serum (ThermoFisher Scientific), 1 % Penicillin/Streptomycin (ThermoFisher Scientific), 20 ng/ml epidermal growth factor (ThermoFisher Scientific), 0.5 mg/ml hydrocortisone (Sigma Aldrich), 100 ng/ml cholera toxin (Sigma Aldrich), and 10 μg/ml insulin (Sigma Aldrich). Both MCF-10A and MCF-10AT cells were encapsulated at a density of 100,000 cells/ml. Media for all cell types was replenished every 2–3 days.

### 4.3. Mechanical characterization of hydrogel matrices

Gels were characterized using an Anton-Paar MCR-502e strain-controlled rheometer. The gel volume was cast between 25 mm diameter plates and a gap of ~1.2 mm. Mineral oil was applied to the edges of the plates to minimize solvent evaporation. The storage modulus was measured using a 0.5 % strain and a frequency of 1 Hz until it reached a steady-state value. For measuring stress relaxation, a 10 % strain was applied, and the time required to reach 50 % of the maximum stress was recorded.

### 4.4. Spheroid invasion assay

Spheroids were formed using the hanging-drop method. Collagen I was added to cell suspensions at a concentration of 6.75 μg/ml 10 μl droplets containing 3000 cells/drop were deposited on Petri dish lids. The lids were then inverted, and the dishes were filled with 1× PBS. Spheroids were allowed to form for 24–36 h. Each spheroid was then collected manually and encapsulated into alginate-collagen matrices. MDA-MB-231 spheroids were cultured in gels for 3 days, while MCF-10A spheroids were cultured for 7 days.

### 4.5. Murine mammary organoid culture

*All procedures were conducted in accordance with the Institutional Animal Care and Use Committee guidelines at the University of California, Santa Barbara.* Mammary organoids were extracted in accordance with a previous protocol published [47]. Briefly, mammary glands from 10-week-old female C57BL/6 strain mice were minced and digested in a collagenase solution on a shaker. 10 ml collagenase solution was prepared by combining 9 ml DMEM/F12 (Fisher #11320082), 0.5 ml fetal bovine serum (Gibco), 5 μl insulin (Sigma, Cat#I-1882), 10 μl gentamicin (50 mg/ml stock, ThermoFisher Cat#15750–060), 200 μl collagenase (100 mg/ml stock, Sigma #2139), and 200 μl trypsin (100 mg/ml stock, Sigma #T7409). The solution was then centrifuged, and the fatty layer was transferred to a BSA-coated tube. The fatty layer was resuspended in DMEM/F12, centrifuged, and the supernatant was discarded. This pellet was combined with the original pellet in a tube and resuspended in 4 ml of DMEM/F12. 40 μl DNase (Sigma Cat#D4263) was added to this suspension, centrifuged, and the supernatant was discarded. The pellet was washed 4× with DMEM/F12 by pulsing it for 3–4s in a centrifuge. Post extraction, organoids were encapsulated in alginate-collagen matrices and maintained in a DMEM/F12 media containing 1 % penicillin/streptomycin, 1 % insulin-transferin-selenium-X (ITS) (Gibco), and 2.5 nM FGF2 (Sigma).

### 4.6. Cell migration assay

MDA-MB-231 cells were stained with 1 μg/ml R18 cell membrane dye (ThermoFisher Scientific) for 1 h before encapsulation into alginate-collagen matrices. Gels containing cells were cast into 8 well glass-bottom live-cell chambers (Labtek). Sterile agarose pieces were used to prevent gels from floating. Live-cell microscopy (Leica SP8 confocal microscope) was performed on day 2 post-encapsulation using a 10× air objective. Cell migration within gels was recorded over a 100 μm thick stack for ~16 h. Maximum Z-projection was performed, and then cell migration average speed and mean-squared displacement were measured using Imaris software. A custom MATLAB script was used to plot individual cell 2D trajectories.

### 4.7. RNA-sequencing and analysis

Cells were extracted from alginate-collagen matrices by rocking them in Falcon tubes containing 2.5 mg/ml collagenase (Sigma, Cat#C0130) solution in PBS for 30 min at 37 °C. After this, the tubes were centrifuged, and the supernatant was removed. Following this, the pellet was dissolved in 10 ml ice-cold EDTA (50 mM) and placed on a rotator for 10 min. The tubes were centrifuged, and the supernatant was then removed. The cell pellet was then lysed using Trizol (Life Technologies), and RNA was extracted using the total RNA mini prep kit according to the manufacturer’s instructions (Epoch Life Sciences).

Bulk-mRNA-sequencing library prep was done using the Cel-Seq2 pipeline as described previously [99,100]. Briefly, 10 ng RNA was reverse-transcribed using the CelSeq2 RT-primer, DTT (0.1 M), dNTPs (New England Biolabs, Cat# N0447l), and Superscript II reverse transcriptase (Invitrogen, Cat# 18064014), followed by second-strand synthesis using RNAseH (ThermoFisher Scientific, Cat# EN0202), E. Coli DNA *Pol*I (Invitrogen, Cat# 18010025), E. Coli ligase (Invitrogen, Cat#18052–019), and the second strand buffer (Invitrogen, #18052–019). After cDNA cleanup using DNA beads (AMPure, Cat# A63882), in vitro transcription was performed using the MEGAscript T7 kit (Invitrogen, Cat# A57622), resulting in amplified single stranded RNA (aRNA). After aRNA treatment with ExoSAP-IT PCR reagent (ThermoFisher, 78200), RNA strands were fragmented using a fragmentation buffer containing 200 mM Tris-acetate (pH 8.1), 500 mM KOAc, and 150 mM MgOAc. aRNA cleanup was then performed using RNA-beads (AMPure, Cat#A63987). Next, aRNA was reverse transcribed using a RT random hex primer, and cDNA was then amplified using an RNA PCR primer (RPI), a uniquely indexed Illumina primer, and a PCR master mix (New England, Cat#M0541S). After bead cleanup and quality control using a bioanalyzer (Agilent), DNA libraries were sequenced using the NovaSeq^™^ 6000 and X platforms (Illumina). The raw reads were then mapped to the hg19 (GRCh37) reference genome.

Differentially expressed genes were determined using the DESeq2 library in R. A minimum log-fold change value of 1 and an adjusted p-value cutoff value of 0.05 were used in the analysis. Gene Set Enrichment Analysis (GSEA) was performed to determine differentially enriched oncogenic pathways within the C6 oncogenic gene sets in the GSEA software. Transcription factor enrichment analysis was performed using the online TRRUST database. Sp1 target genes were chosen from the TRRUST database. The top ten transcription factors that had the highest number of target genes and an adjusted p-value <0.05 were considered. Principal component analysis (PCA) was performed using the PlotPCA function in the DESeq2 library. Sp1 target gene heatmap and hierarchical clustering were performed using the pheatmap library in R. Kaplan-Meier plots were made using the survival and survminer libraries in R.

### 4.8. CUT&Tag-sequencing and analysis

CUT&Tag pipeline developed by Kaya-Okur et al. [61] was employed using the Active Motif CUT&Tag-IT^™^ kit (cat #53176). Briefly, 250,000 cells per sample were washed twice using 1 ml wash buffer (also containing protease inhibitor) at room temperature. A Concanavalin A bead slurry was prepared by dissolving the beads in 1× binding buffer and separating on a magnetic stand. After repeating this step once, the beads were resuspended in 20 μl of binding buffer and then slowly added to the cell suspension and rotated for 10 min. Next, after removing the supernatant on a magnetic stand, cells were suspended in 50 μl of ice-cold Sp1 antibody buffer containing 5 % digitonin, protease inhibitor, and Sp1 primary antibody (Sigma, cat # 07645, 1:50 dilution) and left on a rotator overnight at 4 °C. The next day, after removing the supernatant on a magnetic stand,100 μl of diluted anti-rabbit secondary antibody (1:100) in Dig-Wash buffer (also containing protease inhibitor and 5 % digitonin) was added to each sample. Tubes were placed on a rotator for 60 min at room temperature. After removing the supernatant, cells were washed 2× using the Dig-Wash buffer. Next, the assembled pA-Tn5 transposome enzyme was diluted in the Dig-300 buffer (also containing protease inhibitor) at a 1:100 concentration and incubated for 60 min at room temperature. After washing with 1 ml Dig-300 buffer twice, 125 μl tagmentation buffer (also containing protease inhibitor and digitonin) was added to each sample and incubated for 60 min at 37 ° C. Tagmentation was then immediately stopped by adding 4.2 μl of 0.5 M EDTA, 1.25 μl of 10 % SDS, and 1.1 μl of Proteinase K (10 mg/ml) to each sample and incubated at 55 °C for 60 min. After this, the supernatant was collected using a magnetic stand, and 625 μl DNA purification binding buffer was added to each tube, and the mixture was transferred to a DNA column. After spinning down tubes at 17,000 g for 1 min, the flow through was discarded, and the column was washed at 17,000 g, using DNA purification wash buffer (also containing 80 % ethanol). The DNA was eluted in 35 μl of nuclease-free water. Next, PCR amplification was performed using the Illumina i7 and i5 indexed primers, dNTPs (New England Biolabs, Cat# N0447l), and PCR master mix (New England, Cat# M0541S).

The size distribution of the libraries was quantified on an Agilent Bioanalyzer 2100 and Qubit fluorometer. Libraries were subjected to paired-end 150 bp Illumina sequencing on a Novaseq X platform. Paired- end reads were aligned to the hg38 genome using Bowtie2 v2.3.5, followed by duplicate removal using PICARD. Primary peak summits were identified using MACS2 (q < 0.01), overlapping peaks across replicates were merged, and regions were recentered and uniformly expanded to ± 250 bp around the summit for downstream read counting. Diffbind was then used to build the consensus peak set and obtain read counts for peaks. Normalized Sp1 peak counts were obtained using the edgeR and limma packages (Bioconductor) in R. Heatmaps of normalized peak signal were generated using the pheatmap library. Differential peaks were obtained using the limma package in R. Violin plots for all differential peaks with an adjusted p-value <0.05 were generated using ggplot. Peak annotations were performed using the ChIPseeker and TxDb.Hsapiens.UCSC.hg38.knownGene libraries in R. All genes with differential Sp1 binding log_2_ fold change and RNA-seq gene expression log_2_ fold change, and p-value <0.15 were considered. The ComplexHeatmap library was used to plot heatmaps for these gene subsets. For heatmaps depicting log_2_ fold change in Sp1 binding and the corresponding changes in gene expression, in case of multiple Sp1 binding peaks for a gene, the peak closest to the transcription start site (TSS) was considered.

### 4.9. Patient survival analysis

The microarray and patient clinical data for the low-claudin cohort were obtained from the dataset [60] deposited in the Gene Expression Omnibus (GEO) under the accession number GEO: GSE18229. GPL annotation data was obtained using the GEOquery library (Bio-conductor). Kaplan-Meier plots for genes overlapping with the fast relaxing gene set in this dataset were plotted using a custom code written in R. Plots were made using the survival, survminer, and ggplot2 libraries in R.

### 4.10. Immunofluorescence imaging

Cell culture media was removed and replaced with 4 % paraformaldehyde. After fixing for 45 min at 37 °C, gels were washed 2–3X with DPBS containing Ca^2+^/Mg^2+^. For cells plated on 2D glass, fixation was performed at room temperature for 15 min. Gels were then dehydrated overnight at 4 ° C in a 30 % sucrose solution made in DPBS containing Ca^2+^/Mg^2+^. The next day, gels were incubated in a 1:1 solution of OCT (Fisher Scientific) and 30 % sucrose solution for 4–6 h at 4 ° C. The gels were then frozen in cryomolds in OCT over dry ice. 40 μm thick gel sections were cut using a cryostat (Leica CM1850) and placed on poly-l-lysine coated glass slides.

Sections were washed in DPBS containing Ca^2+^/Mg^2+^ for 1 h at room temperature and then blocked for 1 h at room temperature using a solution of 1× DPBS (with Ca^2+^/Mg^2+^), 1 % bovine serum albumin, 0.1 % Triton X-100, 0.3 M glycine, 10 % goat serum, and 0.05 % sodium azide. Next, sections were incubated with primary antibody solutions overnight at 4 °C. Sections were then washed 3× using a blocking buffer and incubated in Alexa Fluor 488 antibody solution (1:1000 dilution), DAPI (1 μg/ml), and Alexa-Fluor 647 Phalloidin (1:100, ThermoFisher Scientific). Next, slides were washed 3× with the blocking buffer, mounted using Prolong Gold antifade reagent (ThermoFisher Scientific), and their edges were sealed using nail polish. Antibodies used were anti-phospho-Sp1(T453) (Abcam ab59257) and Alexa Fluor 488 goat anti-rabbit IgG (ThermoFisher, Cat#A11998). Samples were imaged using a Leica SP8 confocal microscope.

### 4.11. Collagen fiber dimensions and fiber alignment analysis

Samples were imaged using the 25× objective on a Leica SP8 confocal microscope using the 488 nm laser in the reflectance mode. Fiber dimensions (length and width), as well as fiber orientation relative to cell boundaries or within a region of interest, were analyzed using CT-Fire, a freely available software written in MATLAB script [101]. Fibers selected were within 200 μm of the cell boundary. Fibers oriented at an angle greater than 70° with respect to the cell boundary were categorized as aligned fibers.

### 4.12. Pharmacological inhibition

Small molecule inhibitors were dissolved in dimethyl sulfoxide (DMSO) and diluted in cell culture media. The inhibitors used were ML-7 (25 μM, Cayman Chemical, #11801), Cytochalasin-D (1 μM, Cayman Chemical, #11330), LY294002 (20 μM, Cayman Chemical, #70920), SCH772984 (0.5 μM for MCF-10A cells and 0.1 μM for MDA-MB-231 cells, Cayman Chemical, #19166), and Mithramycin A (50 nM, Cayman Chemical, #11434). Inhibitor concentrations for ML-7 [19], Cytochalasin-D^19^, LY294002 [17,44], mithramycin-A^17^, and SCH772874 [102-104] were derived from previous studies. A DMSO vehicle was used for the control samples.

### 4.13. Image analysis

Cells stained with phalloidin were used to quantify roundness. Images were thresholded and then analyzed using the ImageJ particle analysis feature. For spheroid invasion assays, spheroids were stained with R18 membrane dye, and their invasion boundaries within the matrix were manually traced. The area and circularity of these invasion boundaries were then quantified using ImageJ. For analyzing the nuclear localization of phospho-Sp1, a binary nuclear mask using the DAPI stain was first created via thresholding using a custom MATLAB code. Multiplying the phospho-Sp1 image channel with the nuclear mask resulted in the phospho-Sp1 within the nucleus. We then thresholded these images to obtain the mean nuclear intensity of phospho-Sp1 in the nucleus in ImageJ. Next, a cytoplasmic mask was created by first creating a binary mask using the phalloidin channel and then subtracting the nuclear mask from it. Cytoplasmic phospho-Sp1 was similarly obtained by multiplying this binary cytoplasm mask with the phospho-Sp1 image. Mean intensity of the cytoplasmic phospho-Sp1 images was then calculated using ImageJ. The ratio of the mean nuclear and cytoplasmic intensity of phospho-Sp1 for each image was used to quantify the nuclear localization.

### 4.14. Statistical analysis

All statistical analyses were done using GraphPad (Prism) v.10.2. Specific tests used for analysis can be found in the figure descriptions.

## Supplementary Material

Supplementary Materials

Video 2

Video 3

Video 1

Video 4

Video 5

Video 6

Video 8

Video 7

## Data Availability

RNA-seq and CUT&Tag-seq datasets generated in this study have been deposited at Dryad and can be accessed at https://doi.org/10.5061/dryad.dfn2z35fm. Additional datasets are available from the corresponding author upon reasonable request.

## Appendix A. Supplementary data

Supplementary data to this article can be found online at https://doi.org/10.1016/j.biomaterials.2025.123755.
