# Supplementary material for: Sp1 mechanotransduction regulates breast cancer cell invasion in engineered viscoelastic extracellular matrices
Source: Biomaterials. Author manuscript; Available in PMC 2026 Apr 27. (PMC13120755; doi:10.1016/j.biomaterials.2025.123755)
Supplement: Supplementary Materials [file NIHMS2164403-supplement-Supplementary_Materials.docx]

**Supplementary Table 1:** Alginate-collagen and alginate-rBM gel recipes for soft/stiff, slow/fast relaxing, and Col_low_/Col_high_/rBM conditions

| **Matrix condition** | **Sodium alginate** | **ECM component** | **CaSO_4_** |
| --- | --- | --- | --- |
| Soft-Slow-Col_low_ | 5 mg/ml LF20/40 | 0.5 mg/ml Col I | 2 mM |
| Stiff-Slow-Col_low_ | 5 mg/ml LF20/40 | 0.5 mg/ml Col I | 21 mM |
| Soft-Fast-Col_low_ | 10 mg/ml UPVLVG | 0.5 mg/ml Col I | 5 mM |
| Stiff-Fast-Col_low_ | 10 mg/ml UPVLVG | 0.5 mg/ml Col I | 17 mM |
| Soft-Slow-Col_high_ | 5 mg/ml LF20/40 | 2 mg/ml Col I | 2 mM |
| Stiff-Slow-Col_high_ | 5 mg/ml LF20/40 | 2 mg/ml Col I | 21 mM |
| Soft-Fast-Col_high_ | 10 mg/ml UPVLVG | 2 mg/ml Col I | 2 mM |
| Stiff-Fast-Col_high_ | 10 mg/ml UPVLVG | 2 mg/ml Col I | 10 mM |
| Soft-Slow-rBM | 5 mg/ml LF20/40 | 4.2 mg/ml rBM | 2 mM |
| Stiff-Slow-rBM | 5 mg/ml LF20/40 | 4.2 mg/ml rBM | 21 mM |
| Soft-Fast-rBM | 10 mg/ml UPVLVG | 4.2 mg/ml rBM | 7 mM |
| Stiff-Fast-rBM | 10 mg/ml UPVLVG | 4.2 mg/ml rBM | 25 mM |


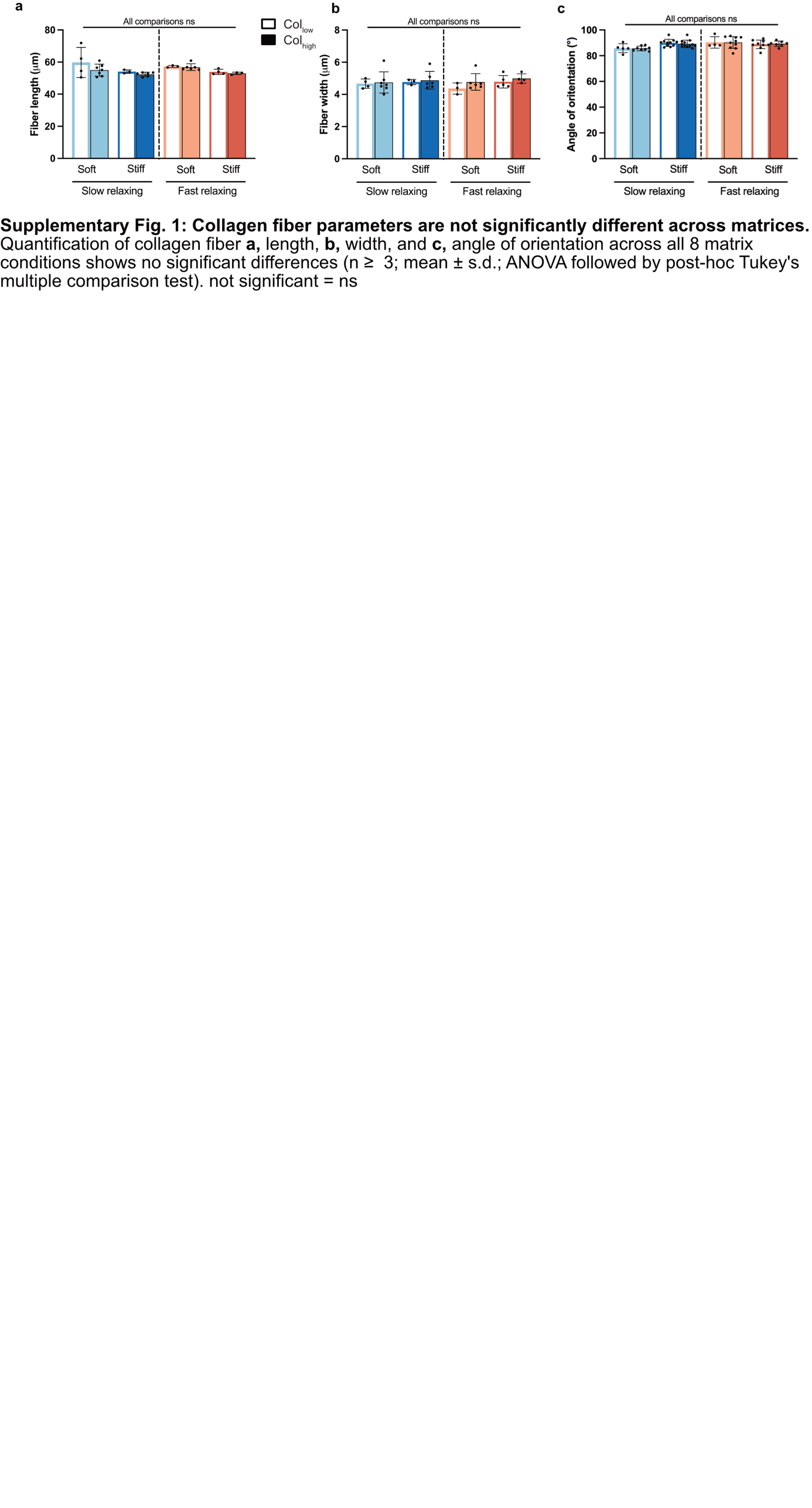
 **Supplementary Fig. 1:** **Collagen fiber parameters are not significantly different across matrices.** Quantification of collagen fiber **a,** length, **b,** width, and **c,** angle of orientation across all 8 matrix conditions shows no significant differences (n ≥ 3; mean ± s.d.; ANOVA followed by post-hoc Tukey's multiple comparison test). not significant = ns


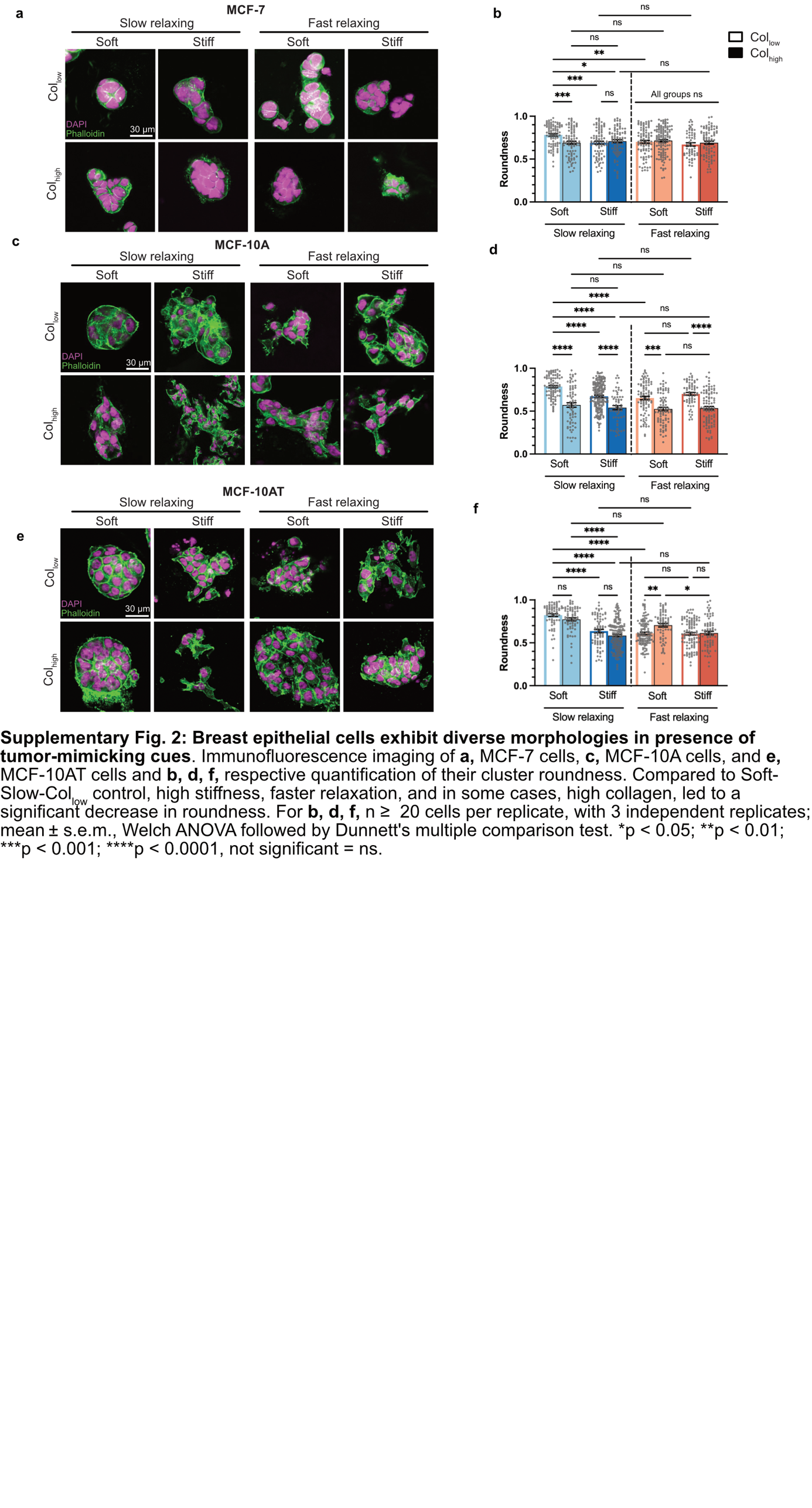


**Supplementary Fig. 2:** **Breast epithelial cells exhibit diverse morphologies in the presence of tumor-mimicking cues**. Confocal immunofluorescence imaging of **a,** MCF-7 cells, **c,** MCF-10A cells, and **e,** MCF-10AT cells and **b, d, f,** respective quantification of their cluster roundness. Compared to Soft-Slow-Col_low_ control, high stiffness, faster relaxation, and in some cases, high collagen, led to a significant decrease in roundness. For **b, d,** **f,** n ≥ 20 cells per replicate, with 3 independent replicates; mean ± s.e.m., Welch ANOVA followed by Dunnett's multiple comparison test. *p < 0.05; **p < 0.01; ***p < 0.001; ****p < 0.0001, not significant = ns.


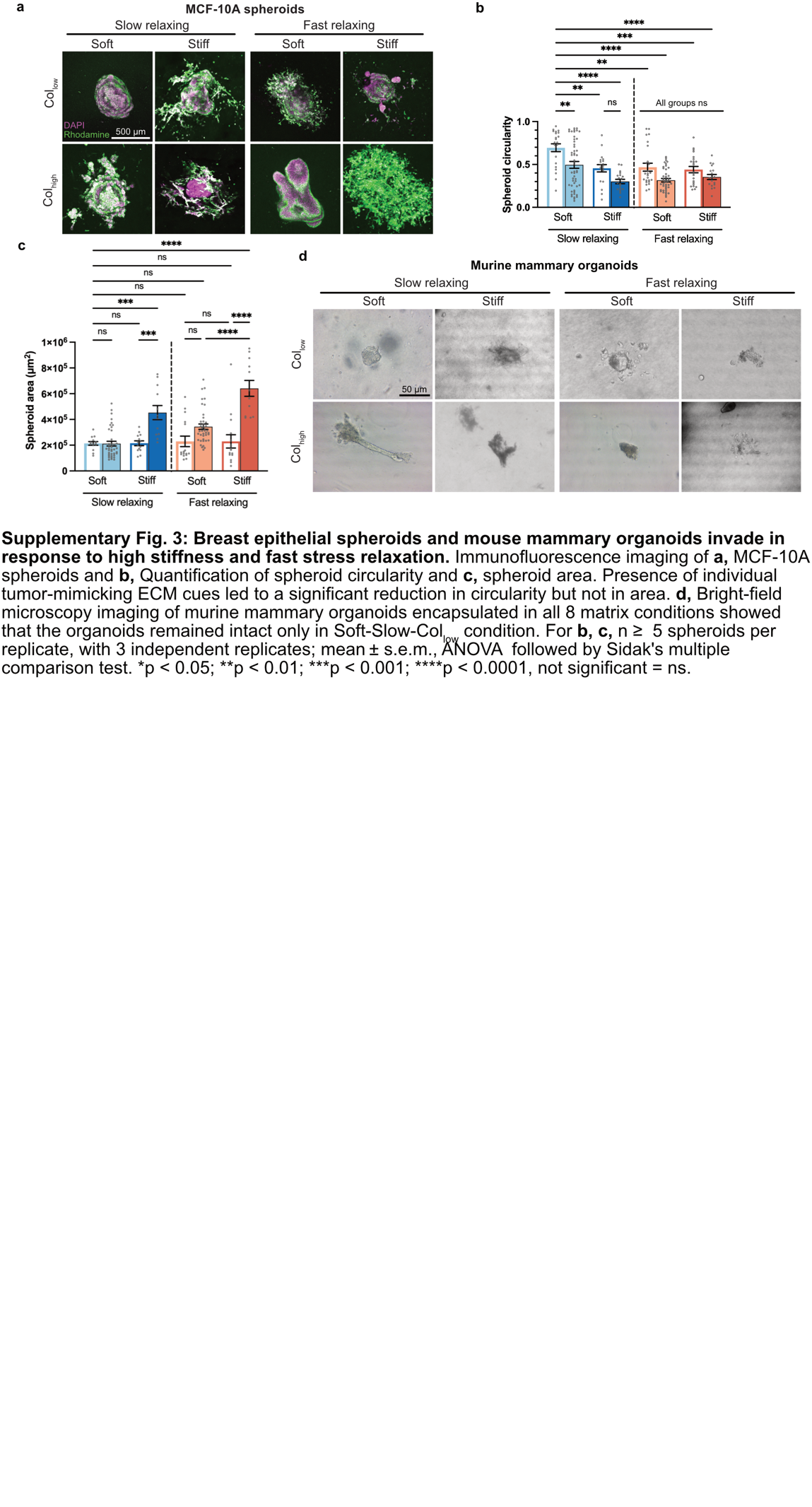


**Supplementary Fig. 3:** **Breast epithelial spheroids and murine mammary organoids invade in response to high stiffness and fast stress relaxation.** Confocal immunofluorescence imaging of **a,** MCF-10A spheroids and **b,** Quantification of spheroid circularity and **c,** spheroid area. The presence of individual tumor-mimicking ECM cues led to a significant reduction in circularity but not in area. **d,** Bright-field microscopy imaging of murine mammary organoids encapsulated in all 8 matrix conditions showed that the organoids remained intact only in Soft-Slow-Col_low_ condition. For **b, c,** n ≥ 5 spheroids per replicate, with 3 independent replicates; mean ± s.e.m., ANOVA followed by Sidak's multiple comparison test. *p < 0.05; **p < 0.01; ***p < 0.001; ****p < 0.0001, not significant = ns.


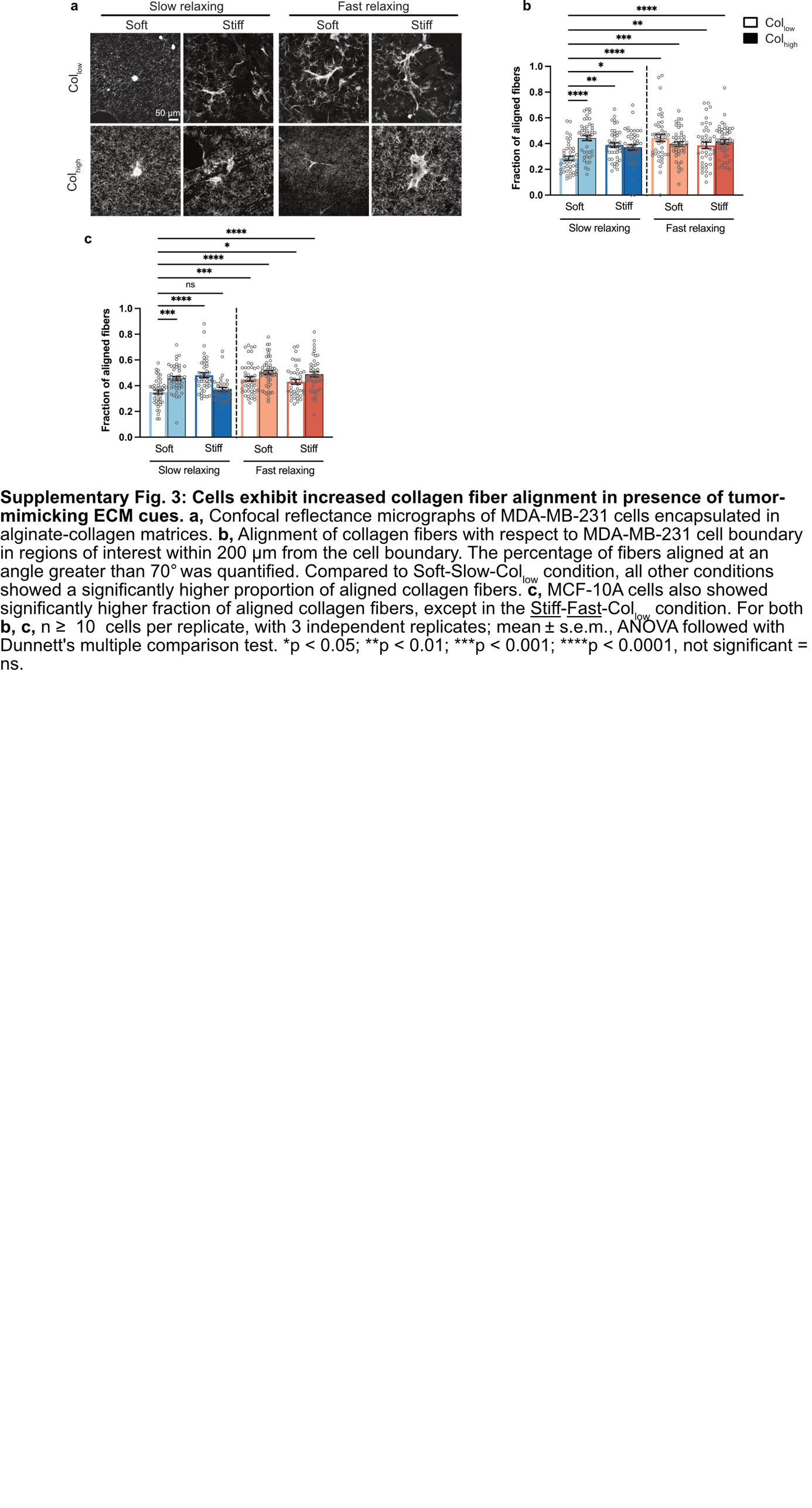


**Supplementary Fig. 4:** **Cells exhibit increased collagen fiber alignment in the presence of tumor-mimicking ECM cues. a,** Confocal reflectance micrographs of MDA-MB-231 cells encapsulated in alginate-collagen matrices. **b,** Alignment of collagen fibers with respect to MDA-MB-231 cell boundary in regions of interest within 200 µm from the cell boundary. The percentage of fibers aligned at an angle greater than 70° was quantified. Compared to Soft-Slow-Col_low_ condition, all other conditions showed a significantly higher proportion of aligned collagen fibers. **c,** MCF-10A cells also showed significantly higher fraction of aligned collagen fibers, except in the Stiff-Fast-Col_low_ condition. For both **b, c,** n ≥ 10 cells per replicate, with 3 independent replicates; mean ± s.e.m., ANOVA followed with Dunnett's multiple comparison test. *p < 0.05; **p < 0.01; ***p < 0.001; ****p < 0.0001, not significant = ns.


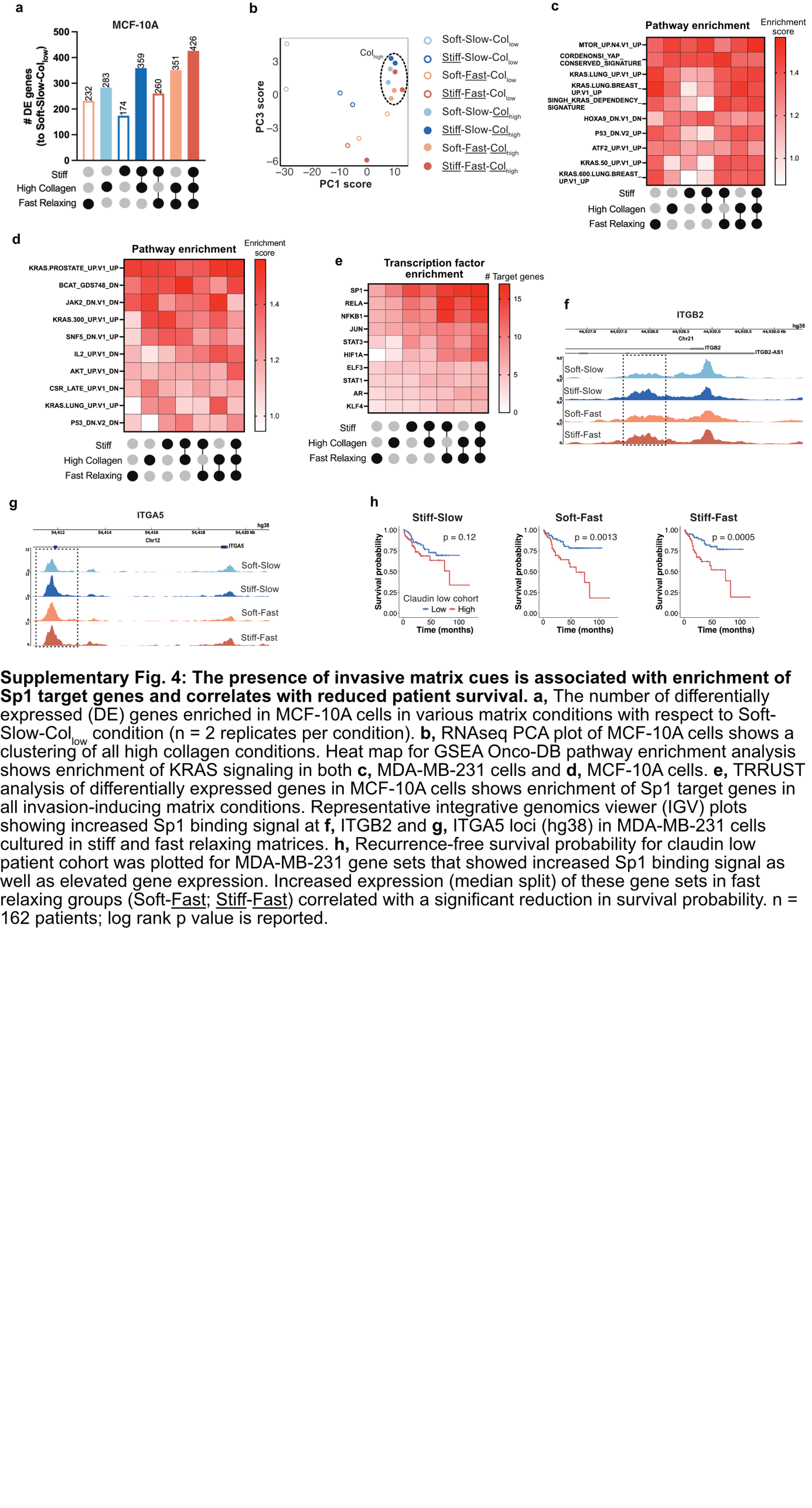


**Supplementary Fig. 5:** **The presence of tumor-mimicking matrix cues is associated with enrichment of Sp1 target genes and correlates with reduced patient survival. a,** The number of differentially expressed (DE) genes enriched in MCF-10A cells in various matrix conditions with respect to Soft-Slow-Col_low_ condition (n = 2 replicates per condition). **b,** RNA-seq PCA plot of MCF-10A cells shows a clustering of all high collagen conditions. **c,** Heat map for GSEA Onco-DB pathway enrichment analysis shows enrichment of KRAS signaling in both MDA-MB-231 cells and **d,** MCF-10A cells. **e,** TRRUST analysis of differentially expressed genes in MCF-10A cells shows enrichment of Sp1 target genes in all invasion-inducing matrix conditions. **f,** Representative integrative genomics viewer (IGV) plots showing increased Sp1 binding signal at ITGB2 and **g,** ITGA5 loci in MDA-MB-231 cells cultured in stiff and fast relaxing matrices. **h,** Recurrence-free survival probability for a claudin-low patient cohort was plotted for MDA-MB-231 gene sets that showed increased Sp1 binding signal as well as elevated gene expression. Increased expression (median split) of these gene sets in fast relaxing groups (Soft-Fast; Stiff-Fast) correlated with a significant reduction in survival probability. n = 162 patients; log rank p value is reported.


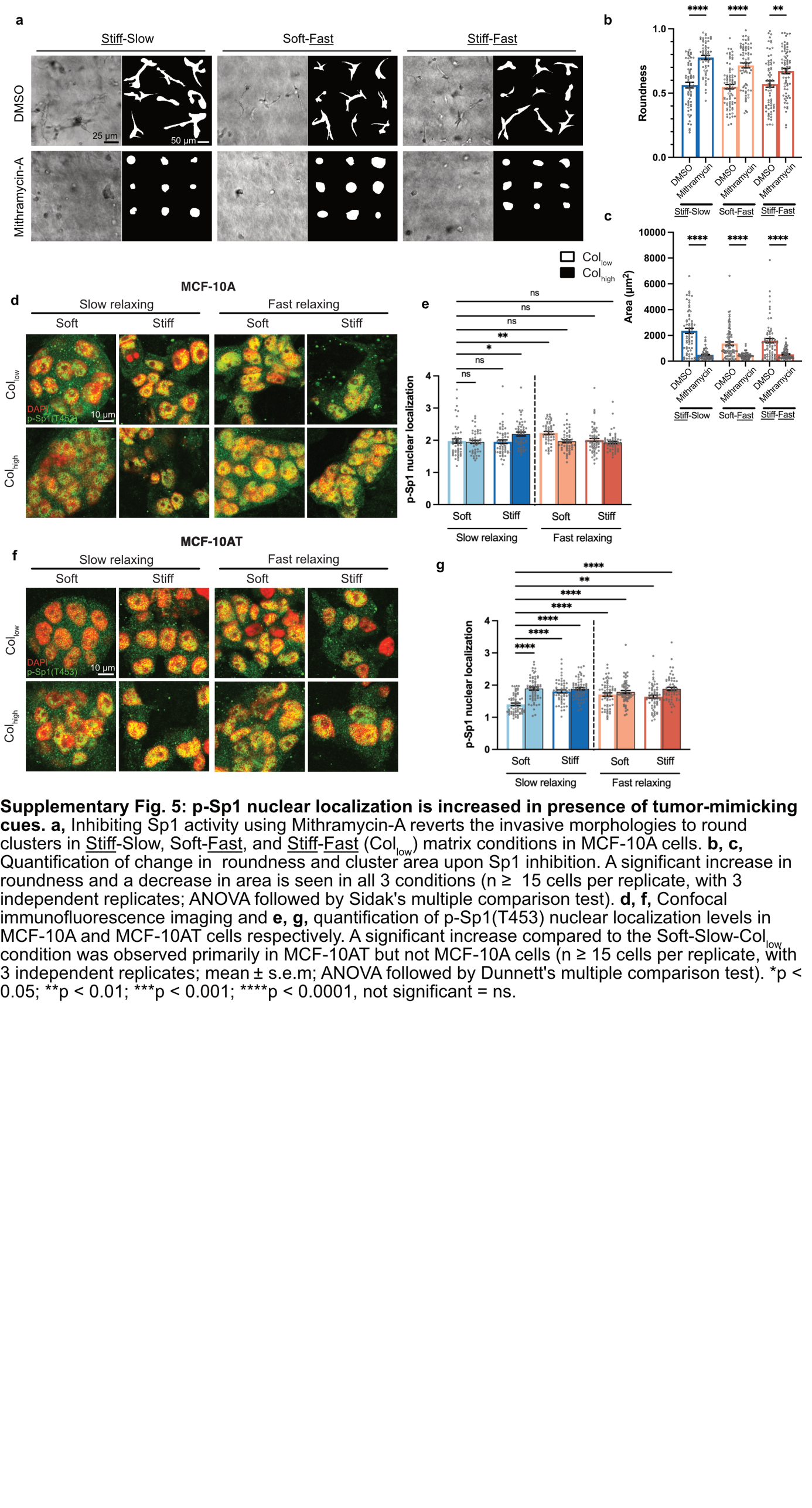


**Supplementary Fig. 6:** **Phospho-Sp1 nuclear localization is increased in the presence of tumor-mimicking cues. a,** Inhibiting Sp1 activity using mithramycin-A reverts the invasive morphologies to round clusters in Stiff-Slow, Soft-Fast, and Stiff-Fast (Col_low_) matrix conditions in MCF-10A cells. **b,** **c,** Quantification of roundness and cluster area upon Sp1 inhibition. A significant increase in roundness and a decrease in area is seen in all 3 conditions (n ≥ 15 cells per replicate, with 3 independent replicates; ANOVA followed by Sidak's multiple comparison test). **d, f,** Confocal immunofluorescence imaging and **e, g,** quantification of phospho-Sp1(T453) nuclear localization levels in MCF-10A and MCF-10AT cells respectively. A significant increase compared to the Soft-Slow-Col_low_ condition was observed primarily in MCF-10AT but not MCF-10A cells (n ≥ 15 cells per replicate, with 3 independent replicates; mean ± s.e.m; ANOVA followed by Dunnett's multiple comparison test). *p < 0.05; **p < 0.01; ***p < 0.001; ****p < 0.0001, not significant = ns.


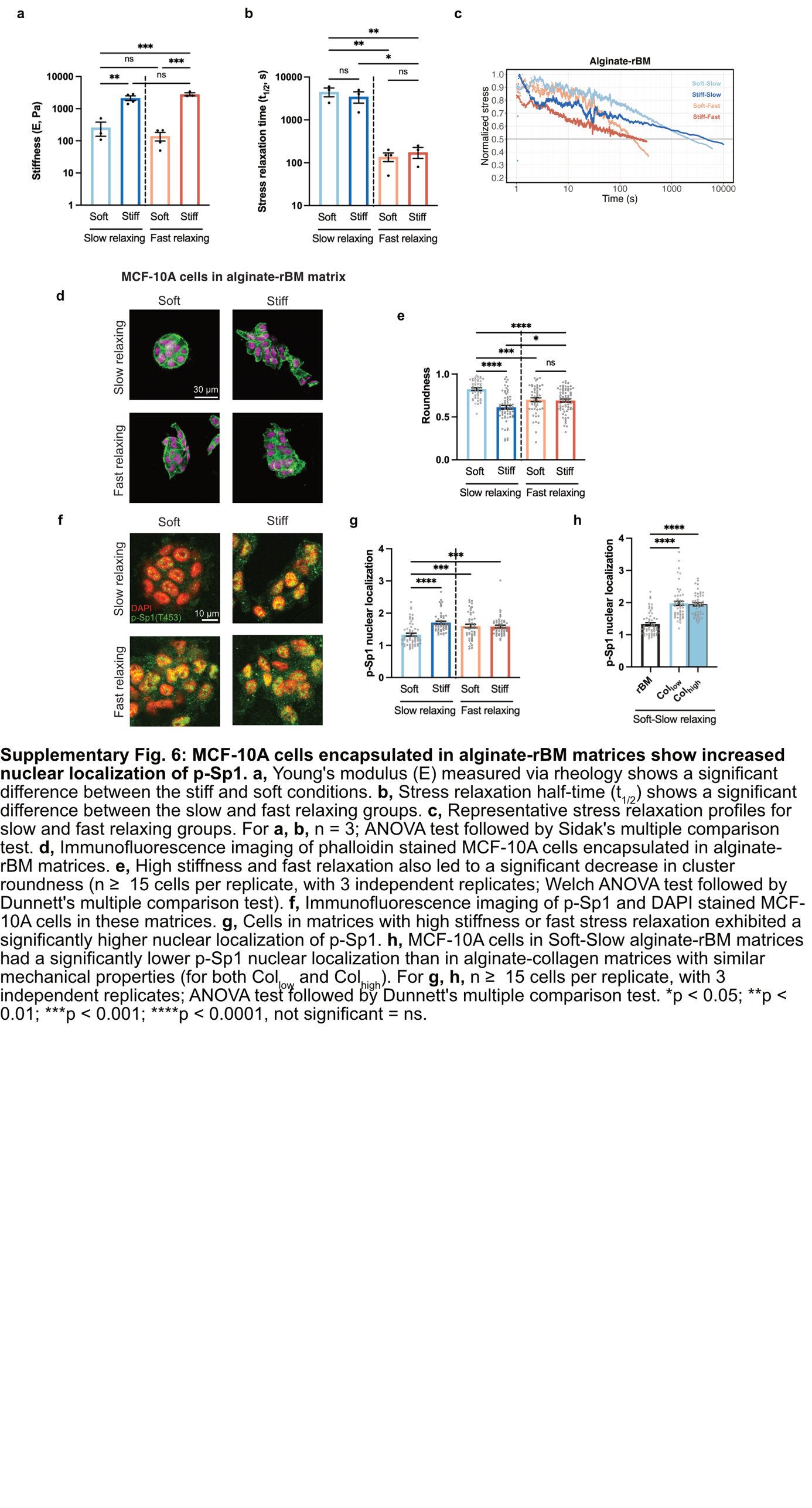


**Supplementary Fig. 7: MCF-10A cells encapsulated in alginate-rBM matrices show increased nuclear localization of phospho-Sp1. a,** Young's modulus (E), estimated from storage and loss moduli measured by rheology, were significantly different between the stiff and soft conditions. **b,** Stress relaxation half-time (t_1/2_) were significantly different between the slow and fast relaxing groups. **c,** Representative stress relaxation profiles for slow and fast relaxing groups. For **a, b,** n = 3; ANOVA test followed by Sidak's multiple comparison test. **d,** Confocal immunofluorescence imaging of phalloidin stained MCF-10A cells encapsulated in alginate-rBM matrices. **e,** High stiffness, and fast relaxation also led to a significant decrease in cluster roundness (n ≥ 15 cells per replicate, with 3 independent replicates; Welch ANOVA test followed by Dunnett's multiple comparison test). **f,** Confocal immunofluorescence imaging of phospho-Sp1 and DAPI stained MCF-10A cells in these matrices. **g,** Cells in matrices with high stiffness or fast stress relaxation exhibited a significantly higher nuclear localization of phosphor-Sp1. **h,** MCF-10A cells in Soft-Slow alginate-rBM matrices had a significantly lower phospho-Sp1 nuclear localization than in alginate-collagen matrices with similar mechanical properties (for both Col_low_ and Col_high_). For **g, h,** n ≥ 15 cells per replicate, with 3 independent replicates; ANOVA test followed by Dunnett's multiple comparison test. *p < 0.05; **p < 0.01; ***p < 0.001; ****p < 0.0001, not significant = ns.


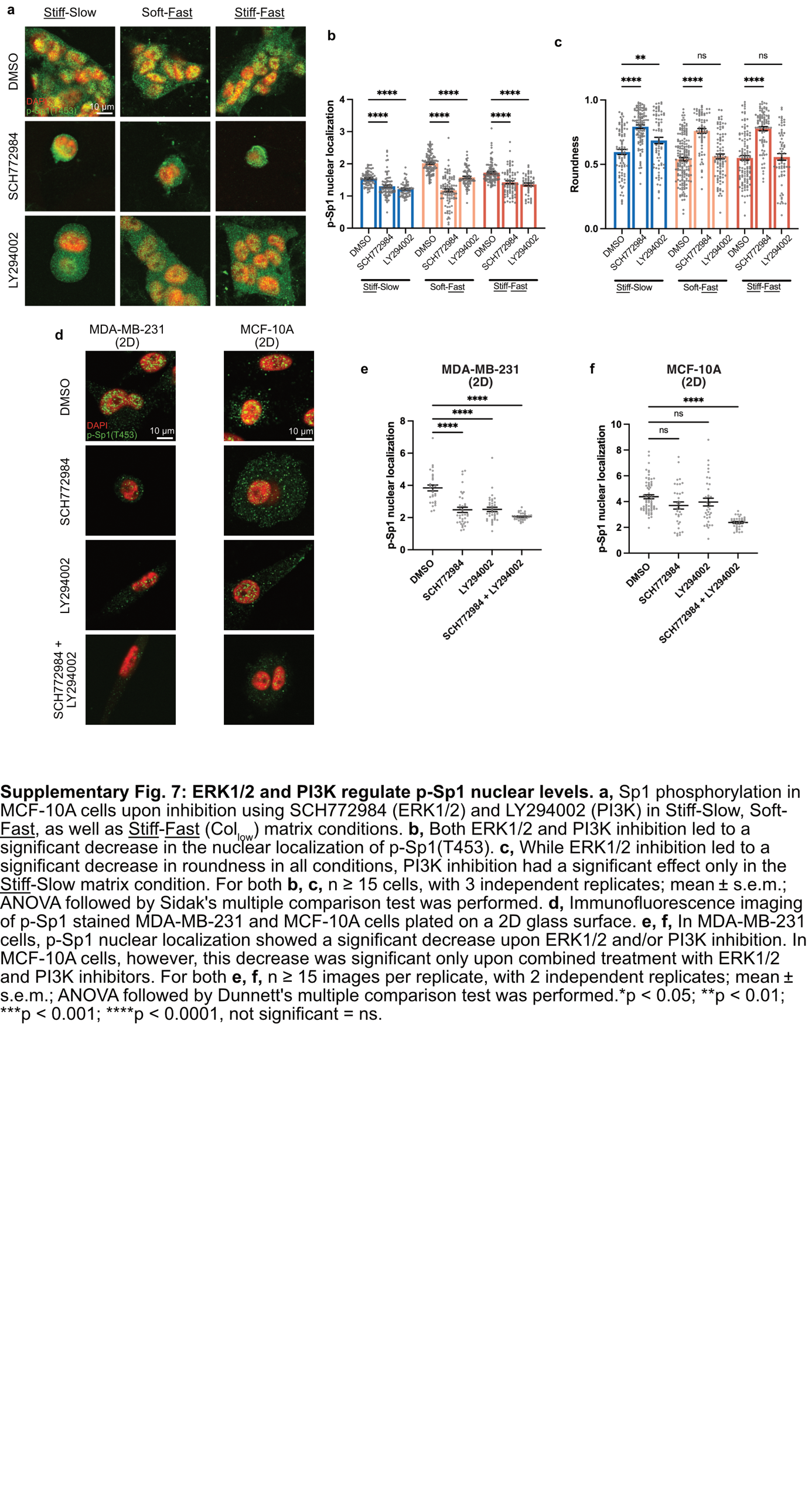


**Supplementary Fig. 8: ERK1/2 and PI3K regulate phospho-Sp1 nuclear levels. a,** Sp1 phosphorylation in MCF-10A cells upon inhibition using SCH772984 (ERK1/2) and LY294002 (PI3K) in Stiff-Slow, Soft-Fast, as well as Stiff-Fast (Col_low_) matrix conditions. **b,** Both ERK1/2 and PI3K inhibition led to a significant decrease in the nuclear localization of phospho-Sp1(T453). **c,** While ERK1/2 inhibition led to a significant decrease in roundness in all conditions, PI3K inhibition had a significant effect only in the Stiff-Slow matrix condition. For both **b, c,** n ≥ 15 cells, with 3 independent replicates; mean ± s.e.m.; ANOVA followed by Sidak's multiple comparison test was performed. **d,** Confocal immunofluorescence imaging of phospho-Sp1 stained MDA-MB-231 and MCF-10A cells plated on a 2D glass surface. **e, f,** In MDA-MB-231 cells, phospho-Sp1 nuclear localization showed a significant decrease upon ERK1/2 and/or PI3K inhibition. In MCF-10A cells, however, this decrease was significant only upon combined treatment with ERK1/2 and PI3K inhibitors. For both **e, f,** n ≥ 15 images per replicate, with 2 independent replicates; mean ± s.e.m.; ANOVA followed by Dunnett's multiple comparison test was performed. *p < 0.05; **p < 0.01; ***p < 0.001; ****p < 0.0001, not significant = ns.


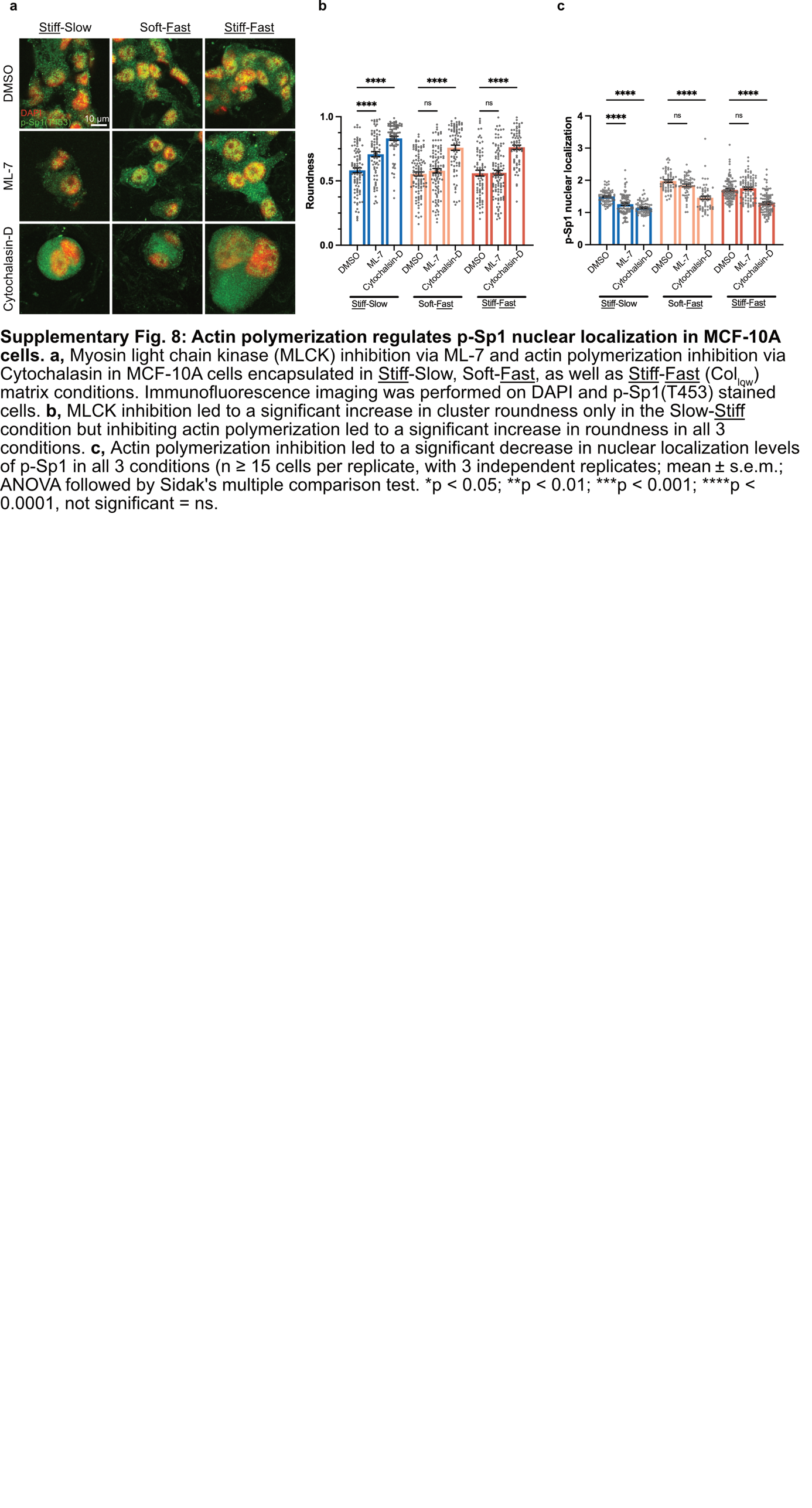


**Supplementary Fig. 9: Actin polymerization regulates phospho-Sp1 nuclear localization in MCF-10A cells. a,** Myosin light chain kinase (MLCK) inhibition via ML-7 and actin polymerization inhibition via Cytochalasin in MCF-10A cells encapsulated in Stiff-Slow, Soft-Fast, as well as Stiff-Fast (Col_low_) matrix conditions. Confocal immunofluorescence imaging was performed on DAPI and phospho-Sp1(T453) stained cells. **b,** MLCK inhibition led to a significant increase in cluster roundness only in the Slow-Stiff condition but inhibiting actin polymerization led to a significant increase in roundness in all 3 conditions. **c,** Actin polymerization inhibition led to a significant decrease in nuclear localization levels of phospho-Sp1 in all 3 conditions (n ≥ 15 cells per replicate, with 3 independent replicates; mean ± s.e.m.; ANOVA followed by Sidak's multiple comparison test. *p < 0.05; **p < 0.01; ***p < 0.001; ****p < 0.0001, not significant = ns.
